# Molecular phylogeny of the Orthalicoidea land snails: Further support and surprises

**DOI:** 10.1371/journal.pone.0288533

**Published:** 2023-07-26

**Authors:** Rodrigo B. Salvador, Fernanda S. Silva, Daniel C. Cavallari, Frank Köhler, John Slapcinsky, Abraham S. H. Breure

**Affiliations:** 1 Faculty of Biosciences, Department of Arctic and Marine Biology, Fisheries and Economics, UiT–The Arctic University of Norway, Tromsø, Norway; 2 The Arctic University Museum of Norway, UiT–The Arctic University of Norway, Tromsø, Norway; 3 Museum of New Zealand Te Papa Tongarewa, Wellington, New Zealand; 4 Museu de Zoologia da Universidade de São Paulo, São Paulo, SP, Brazil; 5 Faculdade de Filosofia, Departamento de Biologia, Ciências e Letras de Ribeirão Preto, Universidade de São Paulo, Ribeirão Preto, SP, Brazil; 6 Australian Museum, Sydney, NSW, Australia; 7 Florida Museum of Natural History, Gainesville, FL, United States of America; 8 Royal Belgian Institute of Natural Sciences, Brussels, Belgium; 9 Department of Life Sciences, Invertebrate Division, Natural History Museum, London, United Kingdom; 10 Naturalis Biodiversity Center, Leiden, The Netherlands; Nanjing Agricultural University, CHINA

## Abstract

The superfamily Orthalicoidea comprises approximately 2,000 species of terrestrial gastropods, mostly concentrated in the Neotropics but also present in southern Africa and Oceania. We provide a multi-marker molecular phylogeny of this superfamily, reassessing its family- and genus-level classification. We exclude two families from the group, Odontostomidae and Vidaliellidae, transferring them to Rhytidoidea based on their phylogenetic relationships as recovered herein. Two new families are recognized herein as members of Orthalicoidea, Tomogeridae and Cyclodontinidae fam. nov. The family Megaspiridae and the subfamily Prestonellinae are paraphyletic but are retained herein for taxonomic stability. The subfamily Placostylinae is synonymized with Bothriembryontinae. The new genera *Alterorhinus* gen. nov. and *Sanniostracus* gen. nov. containing some Brazilian species are described here to better reflect the phylogeny. The fossil record and paleobiogeographic history of the group is explored under the new phylogenetic framework.

## Introduction

The superfamily Orthalicoidea Martens, 1860 contains a diverse assemblage of mostly neotropical snails, conservatively estimated at around 2,000 species [1]. Generally referred to as “tree snails”, their centre of diversity is in the Americas, where they are distributed from southern USA to Patagonia. One lineage is present in Oceania, with most representatives in Australia and several additional species found on the southern Pacific Islands, including Aotearoa New Zealand, the Solomon Islands, Fiji and Vanuatu. Finally, there is also a lineage in southern Africa, with few representatives.

Presently, Orthalicoidea contains seven families [2]: Amphibulimidae Fischer, 1873, Bothriembryontidae Iredale, 1937 (the only one with representatives outside the Americas), Bulimulidae Tryon, 1867, Megaspiridae Pilsbry, 1904, Odontostomidae Pilsbry & Vanatta, 1898, Orthalicidae Martens, 1860, and Simpulopsidae Schileyko, 1999. Recently, the extinct family Vidaliellidae Nordsieck, 1986, from northern Africa and Europe, has been placed within Orthalicoidea [3] based on conchological similarities and without considerations of biogeography or chronology. The fossil record of the Orthalicoidea dates back to the Late Cretaceous in southern South America [4,5].

The studies of Breure et al. [6] and Breure & Romero [7] represented the first attempts to improve our understanding of the principal evolutionary relationships within the Orthalicoidea by means of molecular phylogeneetics. The latter study has been the main source of information underpinning the current classification scheme proposed by [2]. However influential these two studies were in shaping our understanding of orthalicoid relationships, they suffered from poor taxon sampling, a problem that is not unexpected when dealing with such a diverse superfamily from the Global South. The limited taxon sampling, limited resolution and frequently poor branch support for phylogenetic splits has revealed some uncertainties in the taxonomic classification of the Orthalicoidea. Additional molecular studies published after [7], have usually focussed on more specific systematic problems, typically singling out a certain genus of interest (e.g., [8]: *Bulimulus*; [9]: *Clessinia*; [10]: *Hyperaulax*; [11]: *Placostylus*). Yet, no other study has aimed at improving the tree of the Orthalicoidea overall to facilitate a more objective classification of this group.

Here, we are adding new sequences for 80 additional taxa to the available sequence dataset from previously published works, including species from the entire geographic range of the superfamily and from all its major branches. Our goal is to produce a more complete and more representative phylogenetic framework of the Orthalicoidea in their entirety. Based on this phylogeny, we re-assess the family and genus level classification of Orthalicoidea. We exclude two families from the Orthalicoidea, retain one non-monophyletic family for the sake of taxonomic continuity, and recognise two new families, one of which is newly described.

## Material and methods

The specimens used in the present study were obtained from natural history collections in the Americas, South Africa, and Australasia, consisting in whole animals preserved in ethanol 70–98%. The material came from the malacological collections of the following institutions: Academy of Natural Sciences of Drexel University (ANSP, Philadelphia, USA); Australian Museum (AM, Sydney, Australia); Faculdade de Filosofia, Ciências e Letras de Ribeirão Preto (CMRP, Ribeirão Preto, Brazil); Field Museum of Natural History (FMNH, Chicago, USA); Kwa-Zulu Natal Museum (NMSA, Pietermaritzburg, South Africa); Museum of New Zealand Te Papa Tongarewa (NMNZ, Wellington, New Zealand); Museu de Zoologia da Universidade de São Paulo (MZSP, São Paulo, Brazil); Florida Museum of Natural History (UF, Gainesville, USA).

A complete list of the samples used is given in Table 1. The identity of all species was reassessed by the present authors based on current taxonomical literature and comparative material housed in those collections. The exception were the two species in the slug genus *Pellicula*, in which the specific identity could not be satisfactorily assessed and the information on the museum’s labels was trusted.

**Table 1:** Species sequenced for the present study, with information on the GenBank accession numbers, locality where the specimens were collected, and registration number of the voucher specimens in the respective collections (see Material & Methods for the institutions’ acronyms).

Species	COI	H3	ITS2+28S	Voucher	Provenance
*Catracca uhlei Simone, 2022	OP361014	OP381236	OP355600	MZSP 151871	Brazil, Minas Gerais, Vargem Grande
*Amphibulima pardalina* Guppy, 1868	OP361000	OP381220	OP355588	ANSP A23958	Montserrat, Saint Peter, Hope, Hope Ridge
*Amphibulima patula* (Bruguière, 1792)	OP361001	OP381221	OP355589	UF 415439	Dominica, Pagua Bay
*Anctus laminiferus* (Ancey, 1888)	OP361002	OP381222	OP355590	MZSP 117375	Brazil, Piauí, Regeneração
*Anostoma depressum* Lamarck, 1822	OP361003	OP381223	—	UF 449119	Brazil, Ceará, Ubajara
*Anostoma rossi* Weber, 1925	OP361004	OP381224	OP355591	MZSP 110032	Brazil, Goiás, Planaltina
*Bostryx tumidulus* (L. Pfeiffer, 1842)	—	OP381226	OP355592	UF 455607	Peru, Junín, San Ramón, near Chanchamayo River, Gad Gha Kum Lodge
*Bulimulus corumbaensis* Pilsbry, 1897	OP361007	OP381228	OP355594	MZSP 089106	Brazil, Mato Grosso do Sul, Maracaju
*Bulimulus guadalupensis* (Bruguière, 1789)	OP361008	OP381229	OP355595	FMNH 344969.1	Puerto Rico
*Bulimulus guadalupensis* (Bruguière, 1789)	OP361009	OP381230	OP355596	MZSP 130582	USA, Florida
*Bulimulus tenuissimus* (d’Orbigny, 1835)	OP361006	OP381227	OP355593	MZSP 133135	Brazil, Santa Catarina, margins of Rio Cubatão
*Bulimulus tenuissimus* (d’Orbigny, 1835)	OP361010	OP381231	OP355597	NMNZ M.328497	Brazil, Paraíba, Areia, Centro de Ciências Agrárias
*Bulimulus tenuissimus* (d’Orbigny, 1835)	OP361011	OP381232	OP355598	NMNZ M.328502	Brazil, São Paulo, São Vicente, UNESP university, Biosciences Institute
*Burringtonia exesa* (Spix, 1827)	OP361012	OP381233	—	MZSP 134491	Brazil, Bahia
*Burringtonia labrosa* (Menke, 1828)	OP361013	OP381235	OP355599	MZSP 106663	Brazil, Espírito Santo, Sooretama, Trilha do Jequitibá
*Cochlorina aurisleporis* (Bruguière, 1792)	OP361015	OP381237	OP355601	MZSP 131959	Brazil, São Paulo, Praia Grande
*Cochlorina aurismuris* (S. Moricand, 1838)	—	OP381238	OP355602	MZSP 134489	Brazil, Rio de Janeiro
*Cochlorina involuta* (E. von Martens, 1867)	—	OP381240	OP355603	MZSP 096758	Brazil, Rio de Janeiro, Arraial do Cabo
*Corona regina* (Férussac, 1823)	OP361016	OP381242	OP355604	MZSP 094158	Brazil, Rondônia, Porto Velho, margins of Rio Madeira
*Cyclodontina gemellata* (Ancey, 1904)	OP361017	OP381243	OP355605	MZSP 080558	Brazil, Goiás, Terezópolis de Goiás, Fazenda Santa Barbara
*Cyclodontina tudiculata* (E. von Martens, 1868)	—	OP381244	OP355606	MZSP 032866	Brazil, São Paulo, Sete Barras, Vale do Ribeira, Núcleo Sete Barras
*Diplomorpha delatouri* (Hartman, 1886)	OR148288	OR162354	OR149871	AM 487482	Vanuatu, Espiritu Santo
*Drymaeus* cf. *schadei* Quintana & Magaldi, 1985	OP361022	OP381251	OP355611	NMNZ M.333508	Brazil, São Paulo, Jardinópolis, Rio Pardo
*Drymaeus dominicus* (Reeve, 1850)	OP361018	OP381246	OP355607	UF 449294	USA, Florida, Dade County
*Drymaeus dormani* (W.G. Binney, 1857)	OP361019	OP381248	OP355608	UF 369107	USA, Florida, Gainesville
*Drymaeus elongatus* (Röding, 1789)	—	OP381258	OP355618	ANSP A469939	Puerto Rico, Guánica, off Cueva Trail
*Drymaeus gereti* Ancey, 1901	OP361020	OP381249	OP355609	MZSP 137071	Brazil, Goiás, São Domingos, Angélica Cave
*Drymaeus gereti* Ancey, 1901	OP361021	OP381250	OP355610	CMRP 877	Brazil, São Paulo, Jardinópolis, Rio Pardo
*Drymaeus immaculatus* (C.B. Adams in Reeve, 1850)	OP361023	OP381252	OP355612	ANSP A23822	Jamaica, Saint Ann, Knutsford
*Drymaeus multilineatus* (Say, 1825)	OP361024	OP381253	OP355613	FMNH 381507.1	USA, Florida, Florida City
*Drymaeus papyraceus* (Mawe, 1823)	OP361025	OP381254	OP355614	NMNZ M.328496	Brazil, Paraíba, Areia, Centro de Ciências Agrárias
*Drymaeus poecilus* (d’Orbigny, 1835)	OP361026	OP381255	OP355615	MZSP 108408	Brazil, Mato Grosso, Itiquira
*Drymaeus rufescens* (J.E. Gray, 1825)	OP361027	OP381256	OP355616	UF 271152	Colombia, Providencia Island
*Drymaeus sulphureus* (L. Pfeiffer, 1857)	OP361028	OP381257	OP355617	ANSP A18320	Costa Rica, Limon
*Eumecostylus hargravesi* (Cox, 1871)	OP361029	OP381260	OP355620	ANSP A469203	Solomon Islands, Malaita, Auki
*Eumecostylus hargravesi* (Cox, 1871)	OR148282	OR162348	OR149865	AM 557181	Solomon Islands, Malaita
*Eumecostylus hargravesi* (Cox, 1871)	OR148283	OR162349	OR149866	AM 557157	Solomon Islands, Malaita
*Gaeotis flavolineata* Shuttleworth, 1854	OP361030	OP381261	OP355621	ANSP A24708	Puerto Rico, Río Grande, Bosque Nacional del Caribe (El Verde)
*Hyperaulax rildeyi* (E.A. Smith, 1890)	MN175954†	OP381262	OP355622	MZSP 089940	Brazil, Fernando de Noronha, Mirante
*Kora rupestris* Salvador & Simone, 2016	OP361031	OP381263	OP355623	MZSP 137046	Brazil, Minas Gerais, Parque Nacional Cavernas do Peruaçu, Lapa dos Brancos
*Leiostracus carnavalescus* Simone & Salvador, 2016	OP361032	OP381264	OP355624	MZSP 106177 [holotype]	Brazil, Minas Gerais, Nanuque
*Leiostracus carnavalescus* Simone & Salvador, 2016	OP361033	OP381265	OP355625	MZSP 106178 [paratype]	Brazil, Minas Gerais, Nanuque
*Leiostracus demerarensis* (Pfeiffer, 1861)	MN175958‡	OP381266	OP355626	NMNZ M.328329	Brazil, Pará, Jacareacanga, Thaimaçu Lodge property
*Leiostracus* sp.	OP361034	OP381267	OP355627	MZSP 086773	Brazil, Bahia, Buerarema, Fazenda Tororo
*Leiostracus vittatus* (Spix, 1827)	OP361035	OP381268	OP355628	MZSP 134337	Brazil, Bahia
*Liguus virgineus* (Linnaeus, 1758)	—	OP381269	OP355630	ANSP A16560	Dominican Republic, Santiago, Yaque del Norte River, la Angostura, las Charcas
*Megaspira robusta* Pilsbry, 1904	OP361036	OP381270	OP355631	MZSP 086740	Brazil, Rio de Janeiro, Serra de Macaé
*Moricandia dubiosa* (Jay, 1839)	OP361037	OP381271	OP355632	MZSP 096929	Brazil, Espírito Santo, Mimoso do Sul, Conceição
*Odontostomus paulista* Pilsbry & Ihering, 1898	OP361038	OP381273	OP355634	MZSP 090812	Brazil, São Paulo, Peruibe
*Orthalicus floridensis* Pilsbry, 1891	OP361039	—	OP355635	UF 449417	USA, Florida, Homestead
*Orthalicus pulchellus* (Spix, 1827)	OP361040	—	OP355636	CMRP 890	Brazil, São Paulo, Jardinópolis, Rio Pardo
*Orthalicus pulchellus* (Spix, 1827)	—	OP381274	OP355637	CMRP 891	Brazil, São Paulo, Ribeirão Preto, Campus USP-FFCLRP
*Orthalicus reses* (Say, 1830)	OP361041	—	OP355638	UF 449418	USA, Florida, Miami
*Otostomus signatus* (Spix in J.A. Wagner, 1827)	OP361042	OP381275	OP355639	MZSP 099725	Brazil, Bahia, Ilhéus
*Oxychona maculata* Salvador & Cavallari, 2013	—	OP381276	OP355640	MZSP 146248	Brazil, Bahia, São José da Vitória
*Pellicula appendiculata* L. Pfeiffer, 1848	—	OP381277	OP355641	ANSP A24456	Montserrat, Saint Peter, Hope, Hope Ridge
*Pellicula depressa* Rang, 1853	—	OP381278	OP355642	ANSP A23960	Montserrat, Saint Georges, Jack Boy Hill, New Windward Estate
*Peltella iheringi* Leme, 1968	OP361043	OP381279	OP355643	MZSP 139946	Brazil, São Paulo, Cunha
*Placocharis macfarlandi* (Brazier, 1876)	OR148286	OR162352	OR149869	AM 557199	Solomon Islands, Malaita
*Placocharis malaitensis* (Clench, 1941)	OR148284	OR162350	OR149867	AM 557158	Solomon Islands, Malaita
*Placocharis ophir* (Clench, 1941)	OR148285	OR162351	OR149868	AM 557183	Solomon Islands, Malaita
*Placocharis ophir* (Clench, 1941)	OR148287	OR162353	OR149870	AM 557156	Solomon Islands, Malaita
*Plectostylus broderipii* (G.B. Sowerby I, 1832)	OP361044	OP381280	OP355644	FMNH 331458.1	Chile, Los Ríos, Valdivia, Mehuin
*Plectostylus peruvianus* (Bruguière, 1789)	—	OP381281	OP355645	FMNH 331459.1	Chile, Los Lagos, Llanquihue, Lago Chapo
*Plekocheilus lacerta* (L. Pfeiffer, 1855)	OP361045	OP381259	OP355619	MZSP 147234	Brazil, Pará, Belém
*Plekocheilus nebulosus* Breure, 2009	OP361046	OP381282	OP355646	MZSP 089698	Brazil, Amazonas, São Gabriel da Cachoeira, Pico da Neblina
*Prestonella nuptialis* (Melvill & Ponsonby, 1894)	OP361047	OP381283	OP355647	NMSA-Mol 0W8664	South Africa
*Pseudoxychona dulcis* (Ihering, 1912)	—	OP381284	OP355648	MZSP 108880	Brazil, Espírito Santo, Sooretama
*Pseudoxychona pileiformis* (Moricand, 1836)	—	OP381285	—	MZSP 142821	Brazil, Espírito Santo, Aracruz
*Rabdotus levis* (Dall, 1893)	OP361049	OP381286	OP355649	FMNH 344971.1	Mexico, Baja California Sur, Todos Santos
*Rhinus botocudus* Simone & Salvador, 2016	OP361050	OP381287	OP355650	MZSP 106174 [holotype]	Brazil, Minas Gerais, Nanuque
*Rhinus taipuensis* (F. Baker, 1914)	OP361051	OP381288	OP355651	MZSP 089938	Brazil, Rio Grande do Norte, Natal, Ponte Negra
*Simpulopsis* sp.	OP361053	OP381290	OP355653	MZSP 106510	Brazil, Espírito Santo, Iuna, Parque Nacional do Caparaó
*Simpulopsis sulculosa* (Férussac, 1821)	OP361054	OP381289	OP355652	MZSP 140149	Brazil, Paraná, Paranaguá
*Simpulopsis sulculosa* (Férussac, 1821)	OP361052	OP381291	OP355654	MZSP 140149	Brazil, Paraná, Paranaguá
*Simpulopsis tryoni* Pilsbry, 1899	OP361055	OP381292	OP355655	MZSP 106539	Brazil, Espírito Santo, Iuna, Parque Nacional do Caparaó
*Sultana sultana* (Dillwyn, 1817)	OP361056	—	OP355656	MZSP 104240	Brazil, Pará, Altamira, Usina Hidrelétrica de Belo Monte
*Tomigerus clausus* Spix, 1827	OP361057	OP381293	OP355657	MZSP 090952	Brazil, Caeará, Maranguape, Serra de Pacatuba
*Tomigerus corrugatus* Ihering, 1905	MN175956†	OP381294	OP355658	MZSP 043077	Brazil
*Tomigerus pilsbryi* F. Baker, 1914	OP361058	OP381295	—	MZSP 069299	Brazil, Rio Grande do Norte, João Câmara
*Tomigerus pilsbryi* F. Baker, 1914	OP361059	OP381296	OP355659	MZSP 131077	Brazil, Ceará, Santa Quitéria

The * indicates species used as part of the outgroup. The † and the ‡ indicate, respectively, sequences from [10,12].

DNA sequences from a further 86 representatives of the Orthalicoidea were obtained GenBank stemming from the works of [6,7], as well as [9–11,13–17] (Table 2). Representatives of Achatinidae and Strophocheilidae were included as outgroup to root the trees (Tables 1 and 2).

**Table 2:** Further species used in the phylogenetic analysis, with information on GenBank registration numbers, provenance of the sampled animals, and reference to the original publications.

Species	COI	H3	ITS2/28S	Provenance	Reference
*Subulina octona (Bruguière, 1789)	MF415357	MF415329	MF444887	Palau	[13]
*Bahiensis ciaranus* (Dohrn, 1882)	—	JF514702	JF514757	Brazil, São Paulo, Peruíbe	[7]
*Bahiensis punctatissimus* (Lesson, 1830)	—	JF514701	JF514756	Brazil, São Paulo, Peruíbe	[7]
*Bostryx agueroi* Weyrauch 1960	JF514623	JF514667	JF514731	Peru, Lima, between Yauyos and Magdalena	[7]
*Bostryx apodemetes* (d’Orbigny, 1835)	JF514634	JF514678	JF514741	Argentina, Salta, Cerro San Bernardo	[7]
*Bostryx bilineatus* (G.B. Sowerby I, 1833)	JF514637	JF514681	HM027501	Ecuador, Guayas, Isla de Puna	[6,7]
*Bostryx edmundi* Breure & Neubert, 2008	JF514622	JF514666	JF514730	Peru, Lima, between Yauyos and Magdalena	[7]
*Bostryx longispira* Weyrauch 1960	JF514622	JF514666	JF514730	Peru, Lima, between Yauyos and Magdalena	[7]
*Bostryx solutus* (Troschel, 1847)	—	JF514708	JF514764	Peru, Lima, San Mateo	[7]
*Bostryx strobeli* Parodiz, 1956	JF514636	JF514680	HM027498	Argentina, Córdoba, Sierra de Maza	[6,7]
*Bostryx superbus* Weyrauch, 1967	JF514621	JF514665	JF514729	Peru, Lima, Laraos	[7]
*Bostryx torallyi* (d’Orbigny, 1835)	—	JF514709	JF514765	Argentina, Salta, road to Dique Cabra Coral	[7]
*Bothriembryon dux* (L. Pfeiffer, 1861)	JF514643	JF514686	HM027490	Australia, Western Australia, Mt. Caitlin	[6,7]
*Bothriembryon glauerti* Iredale, 1939	MH465634	—	MH465647	Australia	Meinecke et al. (unpublished)
*Bothriembryon whitleyi* Iredale, 1939	MH465633	—	MH465646	Australia	Meinecke et al. (unpublished)
*Bulimulus bonariensis* (Rafinesque, 1833)	JF514632	JF514676	JF514739	USA, Texas, Houston	[7]
*Bulimulus chrysalis* (L. Pfeiffer, 1847)	—	JF514707	JF514763	Guadeloupe, Basse-Terre, Savane a Mulets	[7]
*Bulimulus diaphanus* (L. Pfeiffer, 1855)	JF514633	JF514677	JF514740	Bahamas	[7]
*Bulimulus guadalupensis* (Bruguière, 1789) #3	JF514630	JF514674	JF514738	Dominican Republic, Santo Domingo	[7]
*Bulimulus hummelincki* Breure, 1974	JF514629	JF514673	JF514737	Barbuda, Codrington Village	[7]
*Bulimulus tenuissimus* (d’Orbigny, 1835)	JF514631	JF514675	HM027507	Brazil, Espírito Santo, Vitória	[6,7]
*Clessinia cordovana* (L. Pfeiffer, 1855)	MG963446	—	MH789462	Argentina, Córdoba, Sierras San Marcos	[9]
*Clessinia cuezzoae* (E. Salas, 2010)	MG963442	—	MH789454	Argentina, Córdoba, Cerro de La Cruz	[9]
*Clessinia gracilis* Hylton Scott, 1966	JF514653	—	JF514750	Argentina, Córdoba, Quilpo	[7]
*Clessinia holmbergi* (Parodiz, 1941)	MG963440	—	MH789456	Argentina, Córdoba, Sierras San Marcos	[9]
*Clessinia nattkemperi* (Parodiz, 1944)	MG963438	—	MH789452	Argentina, Catamarca, Pomancillo	[9]
*Clessinia pagoda* Hylton Scott, 1967	JF514613	—	HM027497	Argentina, Córdoba, Quilpo	[6,7]
*Clessinia pagoda* Hylton Scott, 1967 #2	MG963444	—	MH789464	Argentina, Córdoba, Cerro de La Cruz	[9]
*Clessinia pervarians* (Haas, 1936)	JF514614	JF514661	JF514724	Argentina, Córdoba, Sierra de Guasapampa	[7]
*Clessinia philippii* (Doering, 1875)	JF514612	—	JF514723	Argentina, Córdoba, Cruz del Eje	[7]
*Clessinia popana* (Doering, 1877)	JF514616	—	HM027502	Argentina, Córdoba, Inti Huasi-Dean Funes	[6,7]
*Clessinia stelzneri* (Doering, 1875)	JF514617	—	JF514726	Argentina, Córdoba, Dean Funes-Tulumba	[7]
*Clessinia stelzneri* (Doering, 1875) #2	MG963434	—	MH789458	Argentina, Córdoba, Cerro San Vicente	[9]
*Clessinia tucumanensis* (Parodiz, 1941)	JF514615	JF514662	JF514725	Argentina, Tucumán, Vilpos	[7]
*Clessinia tulumbensis* Cuezzo et al., 2018	JF514618	—	JF514727	Argentina, Córdoba, Dean Funes-Tulumba	[7]
*Clessinia tulumbensis* Cuezzo et al., 2018 #2	MG963436	—	MH789460	Argentina, Córdoba, Ruta 16	[9]
*Corona pfeifferi* (Hidalgo, 1869)	JF514654	—	HM027495	Peru, Loreto, Río Curany	[6,7]
*Corona regalis* (Hupé, 1857)	MW033969	MW147744	MW035049	Brazil, Acre, Senador Guiomard, Fazenda Experimetal Catuába	[14]
*Cyclodontina guarani* (d’Orbigny, 1835)	JF514619	JF514663	JF514728	Argentina, Missiones, Puerto Iguazú	[7]
*Discoleus aguirrei* (Doering, 1884)	KT371414	—	KT371388	Argentina [presumably]	[15]
*Discoleus ameghinoi* (Ihering, 1908)	KT371415	JF514698	JF514753	Argentina, Río Negro, Las Grutas [provenance of COI unknown]	[7,15]
*Drymaeus branneri* F. Baker, 1914	—	JF514706	JF514762	Peru, Madre de Dios, Puerto Maldonado	[7]
*Drymaeus elongatus* (Röding, 1789)	—	JF514710	JF514767	Bonaire, Goto Lake	[7]
*Drymaeus expansus* (L. Pfeiffer, 1848)	—	JF514704	JF514760	Peru, Madre de Dios, Reserva Los Amigos	[7]
*Drymaeus inusitatus* (Fulton, 1900)	JF514648	—	HM027503	Costa Rica, Limón, south of Liverpool	[6,7]
*Drymaeus laticinctus* (Guppy, 1868)	JF514646	JF514688	HM027492	Dominica, St. Joseph, Carnholm	[6,7]
*Drymaeus multifasciatus* (Lamarck, 1822)	JF514647	JF514689	JF514747	St. Kitts, Christchurch	[7]
*Drymaeus pamplonensis* Pilsbry, 1939	—	JF514719	JF514768	Colombia, Cauca, Coconuco	[7]
*Drymaeus serratus* (L. Pfeiffer, 1855)	JF514649	JF514690	HM027499	Peru, Huánuco, Tingo Maria	[6,7]
*Drymaeus stramineus* (Guilding, 1824)	—	JF514703	JF514759	St. Vincent, St. David, Trinity Falls trail	[7]
*Drymaeus vexillum* (Broderip, 1832)	JF514625	JF514669	JF514733	Peru, Cajamarca, Puente Munuyac	[7]
*Eumecostylus cleryi* (Petit de la Saussaye, 1850)	MT163274	MT559982	MN567953	Solomon Islands, Guadalcanal	[11]
*Eumecostylus uliginosus* (Kobelt, 1891)	JF514642	JF514685	HM027505	Solomon Islands, Malaita, Rokera	[6,7]
*Gaeotis nigrolineata* Shuttleworth, 1854	JF514659	JF514695	HM027509	Puerto Rico, El Yunque	[6,7]
*Kara thompsonii* (L. Pfeiffer, 1845)	JF514608	JF514713	HM027508	Ecuador, Azuay, San Francisco	[6,7]
*Leiostracus perlucidus* (Spix, 1827)	JF514640	JF514716	JF514744	Brazil, São Paulo	[7]
*Maoristylus ambagiosus* (Suter, 1907)	MT163272	MT559983	MN567951	New Zealand, Far North District	[11]
*Maoristylus hongii* (Lesson, 1830)	MT163273	MT559984	MN567954	New Zealand, Far North District	[11]
*Megaspira elatior* (Spix, 1827)	JF514610	JF514715	JF514721	Brazil, Rio de Janeiro, Serra de Macaé	[7]
*Naesiotus quitensis* (L. Pfeiffer, 1848)	JF514635	JF514679	HM027510	Ecuador, Pichincha, Cayambe	[6,7]
*Naesiotus subcostatus* (Haas, 1948)	JF514652	JF514692	JF514749	Peru, Ancash, Catzcal	[7]
*Orthalicus ponderosus* (Strebel & Pfeffer, 1882)	JF514655	—	HM027506	Mexico, Jalisco, Punta Perula	[6,7]
*Paeniscutalus crenellus* (Philippi, 1867)	JF514609	JF514714	JF514720	Peru, Ancash, Macará	[7]
*Peltella palliolum* (Férussac, 1821)	—	JF514705	JF514761	Brazil, São Paulo, Santo André	[7]
*Pilsbrylia paradoxa* Hylton Scott, 1952	JF514644	JF514687	JF514745	Argentina, Salta, Salta-Jujuy km 1650	[7]
*Placocharis strangei* (L. Pfeiffer, 1855)	JF514641	JF514684	HM027504	Solomon Islands, New Georgia, Munda	[6,7]
*Placostylus fibratus* (Martyn, 1784)	MT163270	MT559980	MN567952	New Caledonia, Isle of Pines	[11]
*Placostylus porphyrostomus* (L. Pfeiffer, 1853)	MT163271	MT559981	MN567955	New Caledonia, Isle of Pines	[11]
*Plagiodontes daedaleus* (Deshayes, 1851)	MG963448	—	MH789466	Argentina, Córdoba, Ruta 16	[9]
*Plagiodontes multiplicatus* (Doering, 1875)	JF514620	JF514664	HM027496	Argentina, Córdoba, Sierra de Cuniputo	[6,7]
*Plagiodontes parvus* Hylton Scott, 1952	—	JF514718	JF514758	Argentina, Córdoba, Cruz del Eje	[7]
*Plagiodontes patagonicus* (d’Orbigny, 1835)	KT371421	—	KT371398	Argentina [presumably]	[15]
*Plectostylus peruvianus* (Bruguière, 1789)	—	JF514697	HM027493	Chile, O’Higgins, Pichilemu	[6,7]
*Plekocheilus breweri* Breure & Schlögl, 2010	JF514657	JF514693	JF514751	Venezuela, Bolívar, Churi-tepui	[7]
*Plekocheilus vlceki* Breure & Schlögl, 2010	JF514658	JF514694	HM027491	Venezuela, Bolívar, Churi-tepui	[6,7]
*Porphyrobaphe iostoma* (G.B. Sowerby I, 1820)	JF514656	—	HM027500	Ecuador, Manabi, Puerto Lopez	[6,7]
*Prestonella bowkeri* (G.B. Sowerby III, 1890)	KF129392	JF514711	EU622021	South Africa, Glen Avon	[7,16]
*Prestonella nuptialis* (Melvill & Ponsonby, 1894)	KF129349	—	EU622022	South Africa, Craddock area	[16,17]
*Protoglyptus luciae* (Pilsbry, 1897)	JF514651	JF514712	JF514748	St. Lucia, Union Quarter	[7]
*Protoglyptus stenogyroides* (Guppy, 1868)	JF514650	JF514691	HM027494	Dominica, St. Georges, path to Lake Boeri	[6,7]
*Rabdotus alternatus* (Say, 1830)	JF514638	JF514682	JF514742	USA, Texas, Santa Ana National Wildlife Refugium	[7]
*Scutalus chiletensis* Weyrauch, 1967	JF514628	JF514672	JF514736	Peru, Cajamarca, Puente Munuyac	[7]
*Simpulopsis decussata* Pfeiffer, 1856	JF514639	JF514683	JF514743	Brazil, São Paulo, Santos	[7]
*Simpulopsis rufovirens* (S. Moricand, 1846)	—	JF514700	JF514755	Brazil, Bahia, Camacã	[7]
*Thaumastus achilles* (Pfeiffer, 1853)	—	JF514699	JF514754	Brazil, Espírito Santo, Guarapari	[7]
*Thaumastus largillierti* (Philippi, 1845)	JF514611	JF514660	JF514722	Brazil, Minas Gerais, Mariana	[7]
*Ventania avellanedae* (Doering, 1881)	KT371413	—	KT371387	Argentina [presumably]	[15]

The * indicates the species used as part of the outgroup.

### DNA extraction and amplification

A small tissue clip of foot muscle of each voucher specimen was used for DNA extraction. The extraction was performed with the QIAGEN DNEasy® Blood & Tissue Kit, following the manufacturer’s standard protocol, with the addition of a repetition of the final step to increase yield. The molecular markers targeted for this study were the same as in [7]: (1) the barcoding fragment of the COI mitochondrial gene, circa 650 bp long, using the primers LCO/HCO [18]; (2) a fragment of the nuclear H3 gene (histone 3), circa 270 bp long, using the primers H3pulF and H3pul3 [19]; (3) a continuous fragment of nuclear DNA including the 3′ end of the 5.8S rRNA gene, the ITS2 region, and the 5′ end of the 28S rRNA gene, totalling circa 1,300 bp long and amplified in two fragments using the primers LSU-1/LSU-3 and LSU-2/LSU-5 [20,21].

The PCR amplification protocols were as follows. COI: initial denaturation at 95°C for 3 min; 35 cycles of denaturation at 95°C for 30 s, annealing at 48°C for 1 min, and extension at 72°C for 2 min; final extension at 72°C for 5 min. COI: initial denaturation at 95°C for 3 min; 40 cycles of denaturation at 95°C for 30 s, annealing at 57°C for 30 s, and extension at 72°C for 40 s; final extension at 72°C for 5 min. ITS2+28S: initial denaturation at 95°C for 3 min; 40 cycles of denaturation at 95°C for 30 s, annealing at either 50°C (ITS2 section) or 45°C (28S section) for 1 min, and extension at 72°C for either 5 min (ITS2 section) or 2 min (28S section); final extension at 72°C for 4 min.

The success of the PCR was visually assessed via agarose gel electrophoresis. The PCR products were cleaned with ExoSAP-IT™ (Affymetrix Inc.) following the manufacturer’s protocol. Samples were prepared and sent to Massey Genome Service (Massey University, Palmerston North, New Zealand) to be Sanger sequenced. Sequences were quality-proofed and de novo assembled in Geneious Prime (v.2020.2.2, Biomatters Ltd.). Consensus sequences were uploaded to GenBank (Table 1).

### Phylogenetic analysis

Sequences were aligned using the MUSCLE plugin [22] in Geneious Prime with default settings (i.e., optimized for accuracy). The resulting alignments were visually proofed for inconsistencies and then run through Gblocks [23] with the least restrictive settings to eliminate poorly-aligned or data-deficient positions that could introduce noise into the analysis. The resulting post-Gblocks alignments were concatenated for a single phylogenetic analysis, with all three genetic markers treated as individual partitions.

Bayesian inference phylogenetic analysis was performed using MrBayes (v.3.2.7, [24]) through the CIPRES Science Gateway (v. 3.3, [25]). Two concurrent analyses, each with 4 Markov chains of 200 million generations (the first 20% discarded as ‘burn-in’), were run with the default priors, nst = 6, rates = invgamma, temperature parameter = 0.1, sampling every 1,000 generations. Substitution model parameters were unlinked across the markers (COI, 16S, and ITS+28S). MCMC convergence was assessed by examining the standard deviation of split frequencies (~0.01) and the potential scale reduction factor, PSRF (~1.0), as well as the trace plots in Geneious [26].

To check for inconsistencies between markers, two further trees were built using only the H3 or the ITS2+28S alignments (species lacking sequences of one of these markers were excluded from both trees; trees not shown). In these cases, only 20 million generations were enough. The COI alignment was not used due to the known low resolution this marker provides to elucidate family level relationships within Orthalicoidea (e.g., [10,12]). Almost no inconsistencies were found, but some interesting differences are discussed below.

Finally, given the unexpected position of two genera (*Odontostomus* and *Pilsbrylia*) outside of Orthalicoidea, a further phylogenetic analysis was conducted to better asses their relationship to other stylommatophorans. This analysis includes a small subset of Orthalicoidea from the analysis above, as well as further taxa chosen to accommodate the potential Helicina superfamilies that these two genera could belong to (Table 3). The same methodology and parameters from above apply, apart from the number of generations, which was set to 80 million.

**Table 3:** Species used in the additional phylogenetic analysis (built to further test the position of *Odontostomus* and *Pilsbrylia*), with information on GenBank registration numbers, provenance of the sampled animals, and reference to the original publications.

Species	COI	H3	ITS2/28S	Provenance	Reference
*Megalobulimus parafragilior* Leme & Indrusiak, 1990	JF514645	JF514717	JF514746	Brazil, São Paulo, Peruíbe	[7]
*Anthinus multicolor* (Rang, 1831)	OP328912	—	OP345022	Brazil, Rio de Janeiro, São Fidélis	This work; voucher MZSP 152925
*Anthinus vailanti* Simone, 2022	—	—	OP345024	Brazil, Minas Gerais, Brasilândia de Minas	This work; voucher MZSP 152118
*Anthinus synchondrus* Simone, 2022	—	—	OP345023	Brazil, Minas Gerais, Unaí, Pedra da Fartura	This work; voucher MZSP 152074
*Anthinus morenus* Simone, 2022	—	—	OP345021	Brazil, Minas Gerais, Paracatu, São José do Sapezal	This work; voucher MZSP 152047
*Catracca uhlei* Simone, 2022	OP328913	OP341594	OP345025	Brazil, Minas Gerais, Brasilândia de Minas	This work; voucher MZSP 151893
*Megalobulimus conicus* (Bequaert, 1948)	MN688645†	—	OP345026	Brazil, Bahia, Cordeiros, Fazenda São João	This work; voucher MZSP 136674
*Megalobulimus conicus* (Bequaert, 1948)	MN756629†	—	OP345028	Brazil, Bahia, Mortungaba	This work; voucher MZSP 143688
*Megalobulimus pergranulatus* (Pilsbry, 1901)	OP328914	—	OP345030	Brazil, São Paulo, Guaratinguetá, Bairro Gomeral	This work; voucher CMRP 889
*Natalina cafra* (Férussac, 1821)	FJ262255	—	FJ262319 + KT970829	South Africa	[27,28]
*Rhytida stephenensis* (Powell, 1930)	MN022733	MN022764	MN022667	New Zealand	[29]
*Megalobulimus oblongus* (O.F. Müller, 1774)	MN022730	MN022761	MN022664	Antigua	[29]
*Dorcasia alexandri* (Gray, 1938)	MN022731	MN022762	MN022665	Namibia, Windhoek	[29]
*Caryodes dufresnii* (Leach, 1815)	MN022732	MN022763	MN022666	Australia, Tasmania, Hobart, Mt. Wellington	[29]
*Albinaria xantostoma* (Boettger, 1883)	MN022724	MN022755	MN022659	Crete	[29]
*Buliminus labrosus* (Olivier, 1804)	MN022723	MN022754	MN022658	Syria, Saladin’s Castle	[29]

The † indicates sequences from [30].

### Nomenclatural acts

The electronic edition of this article conforms to the requirements of the amended International Code of Zoological Nomenclature, and hence the new names contained herein are available under that Code from the electronic edition of this article. This published work and the nomenclatural acts it contains have been registered in ZooBank, the online registration system for the ICZN. The ZooBank LSIDs (Life Science Identifiers) can be resolved and the associated information viewed through any standard web browser by appending the LSID to the prefix "http://zoobank.org/". The LSID for this publication is: urn:lsid:zoobank.org:pub:B5736A88-9939-4D49-B151-8670B2402F2C. The electronic edition of this work was published in a journal with an ISSN, and has been archived and is available from the following digital repositories: PubMed Central, LOCKSS.

## Results

**Fig 1:**
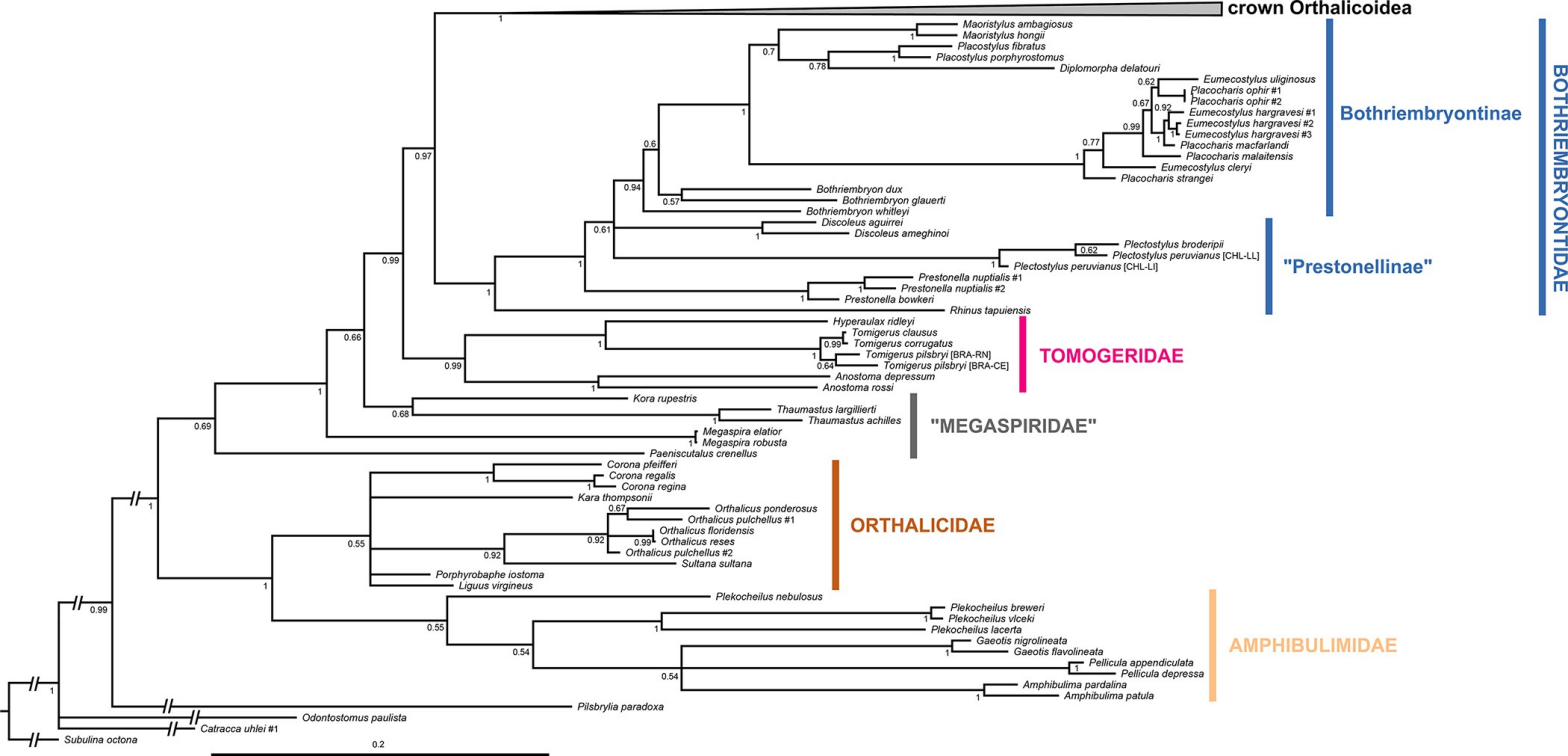
Bayesian inference tree of the Orthalicoidea. The crown group is collapsed to facilitate visualization (see S1 Fig for a full view and Figs 5–7 for the crown Orthalicoidea). Posterior probabilities are shown on nodes. Scale bar is substitutions per site.

**Fig 2:**
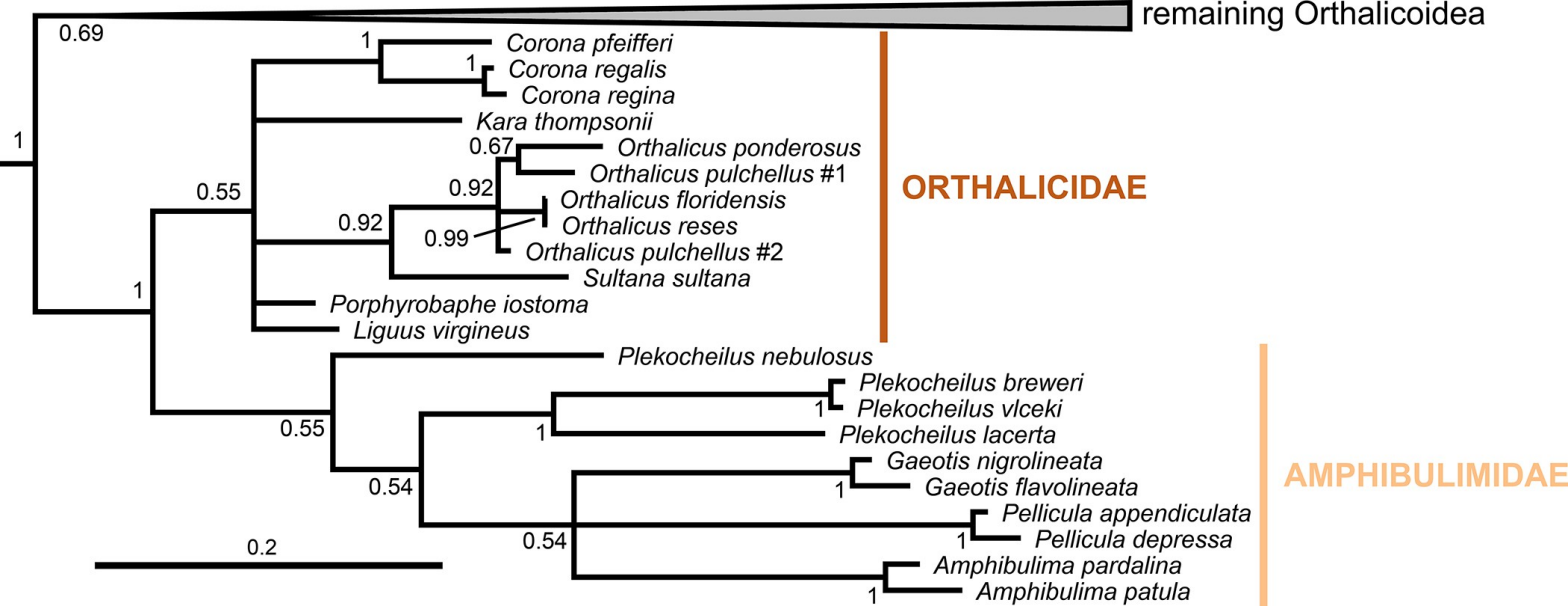
Close-up of Fig 1 showing the Orthalicidae and Amphibulimidae. The remaining Orthalicoidea is collapsed to facilitate visualization (see Fig 1 for a full view) and the outgroup is omitted. Posterior probabilities are shown on nodes. Scale bar is substitutions per site.

**Fig 3:**
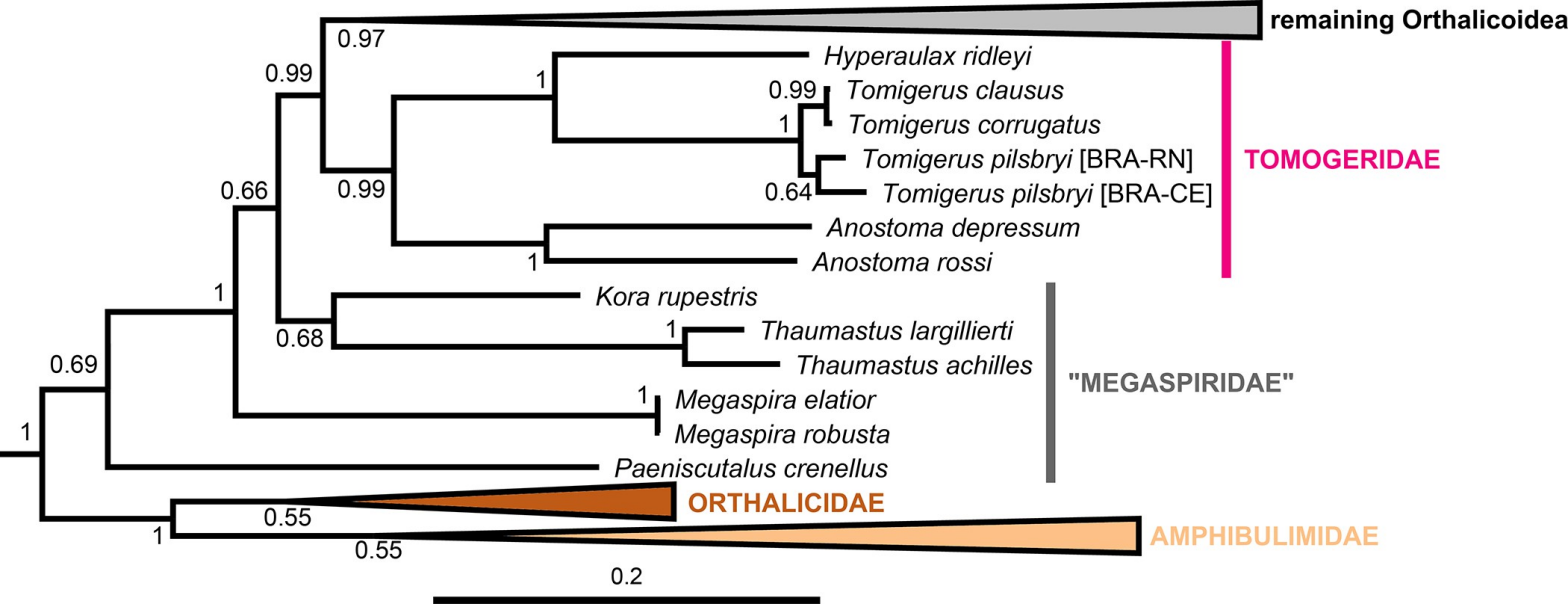
Close-up of Fig 1 showing the “Megaspiridae” and Tomogeridae. The remaining Orthalicoidea is collapsed to facilitate visualization (see Fig 1 for a full view). Posterior probabilities are shown on nodes. Scale bar is substitutions per site.

**Fig 4:**
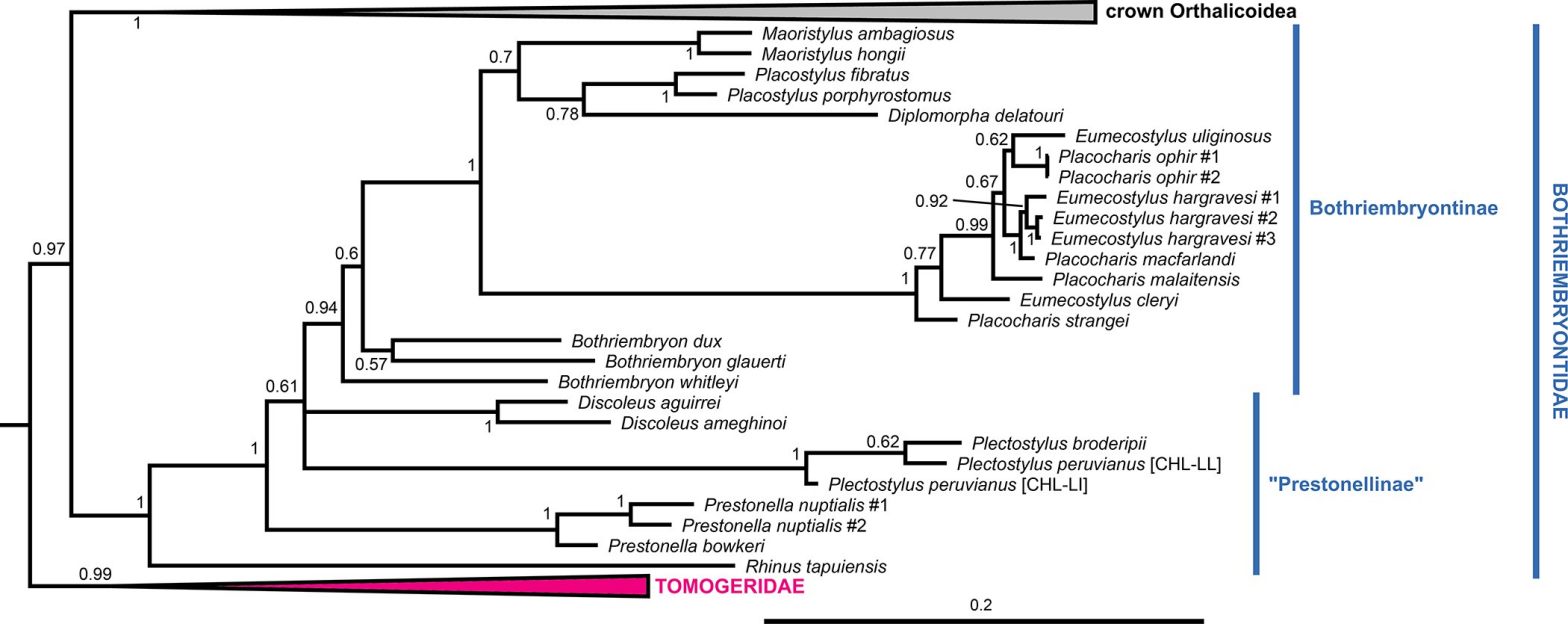
Close-up of Fig 1 showing the Bothriembryontidae. To facilitate visualization, only their sister group, the crown Orthalicoidea (collapsed), are shown (see Fig 1 for a full view). Posterior probabilities are shown on nodes. Scale bar is substitutions per site.

**Fig 5:**
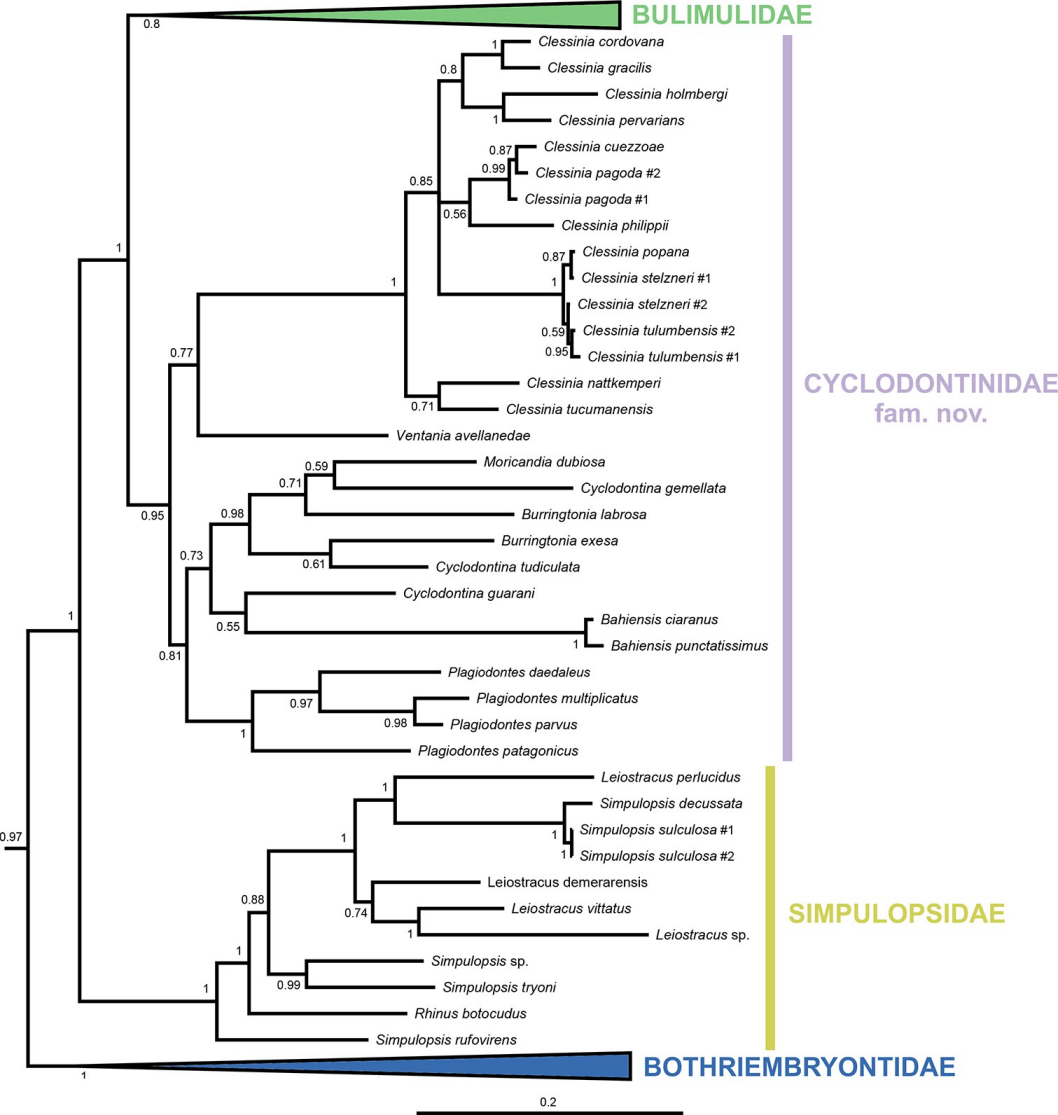
Bayesian inference tree of the crown Orthalicoidea. To facilitate visualization, only their sister group, the Bothriembryontidae (collapsed), are shown, representing the remaining Orthalicoidea (see Fig 1 for a full view); likewise, family Bulimulidae is collapsed (see Fig 6 for a full view). Posterior probabilities are shown on nodes. Scale bar is substitutions per site.

**Fig 6:**
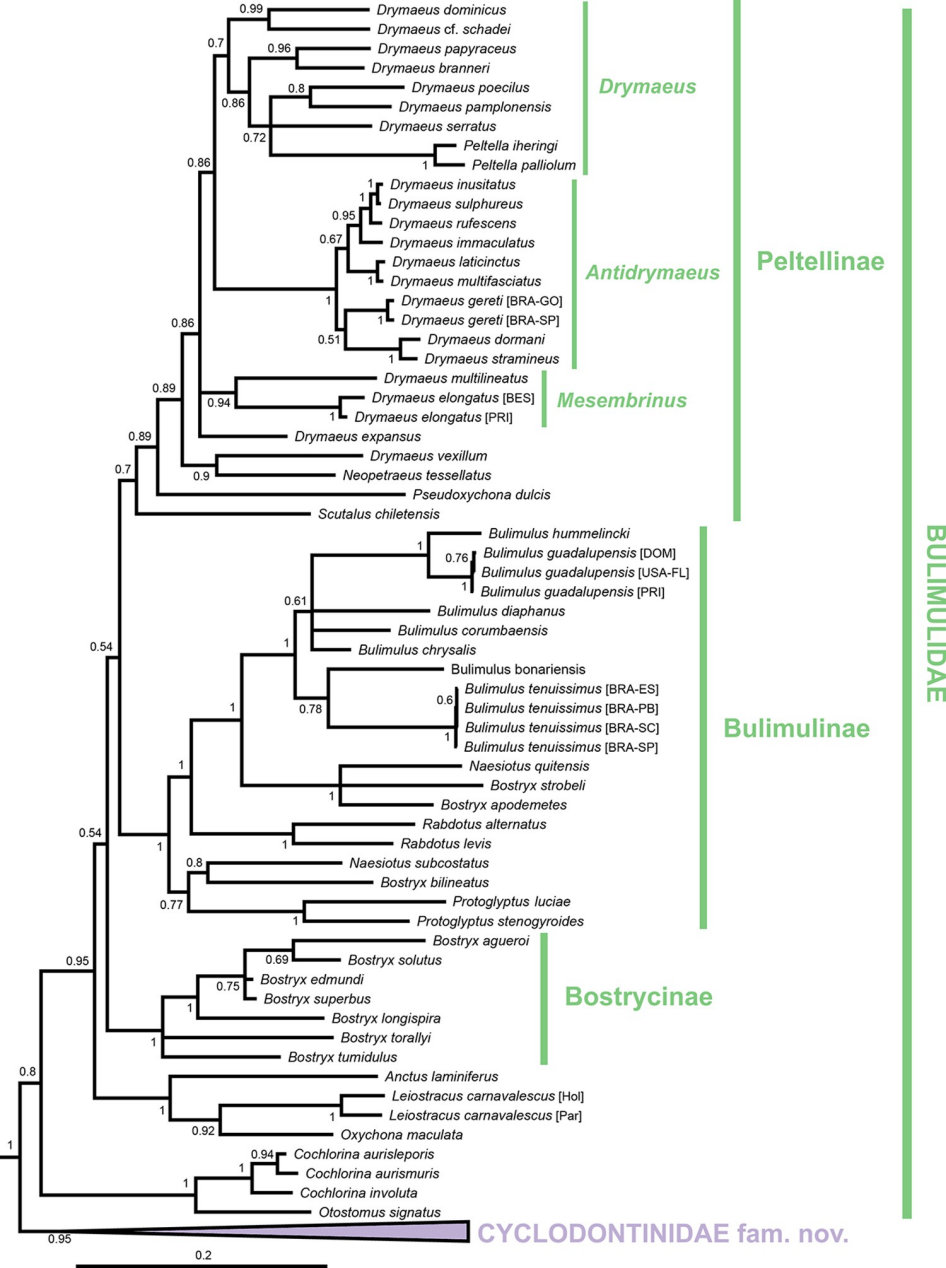
Bayesian inference tree of the Bulimulidae. To facilitate visualization, only their sister group, the Odontostomidae (collapsed), are shown representing the remaining Orthalicoidea (see Figs 1 and 5 for a full view). Posterior probabilities are shown on nodes. Scale bar is substitutions per site. Abbreviations: BES, Bonaire; BRA, Brazil; DOM, Dominican Republic; ES, Espírito Santo state; FL, Florida; GO, Goiás state; Hol, holotype; Par, paratype; PB, Paraíba state; PRI, Puerto Rico; SC, Santa Catarina state; SP, São Paulo state.

The resulting total-evidence tree of the Orthalicoidea contains 171 terminal taxa (including the outgroup), representing 149 species of that superfamily (Figs 1–6 and S1). The concatenated sequences (post trimming with Gblocks) included 1760 bp (COI: 644 bp; H3: 267 bp; ITS2+28S: 849 bp).

The trees built with only one marker (H3 or ITS2+28S) to test for inconsistencies show some loss of resolution, which is to be expected considering these sequences are much shorter and the large number of taxa involved. The loss of resolution is more marked in some species-level relationships (inside *Bulimulus* and *Drymaeus*) and in the appearance of a few family-level polytomies. Nevertheless, almost no inconsistencies were found in relation to the total-evidence tree. The few (and interesting) differences are as follows: (1) in the ITS2+28S tree, the node from which *Odontostomus* branches is more basal than the one from which *Catracca* does; (2) in the H3 tree, there was a better separation of the genera *Leiostracus* and *Simpulopsis* inside family Simpulopsidae, with the former genus being monophyletic, albeit with low support.

### Outgroup

**Fig 7:**
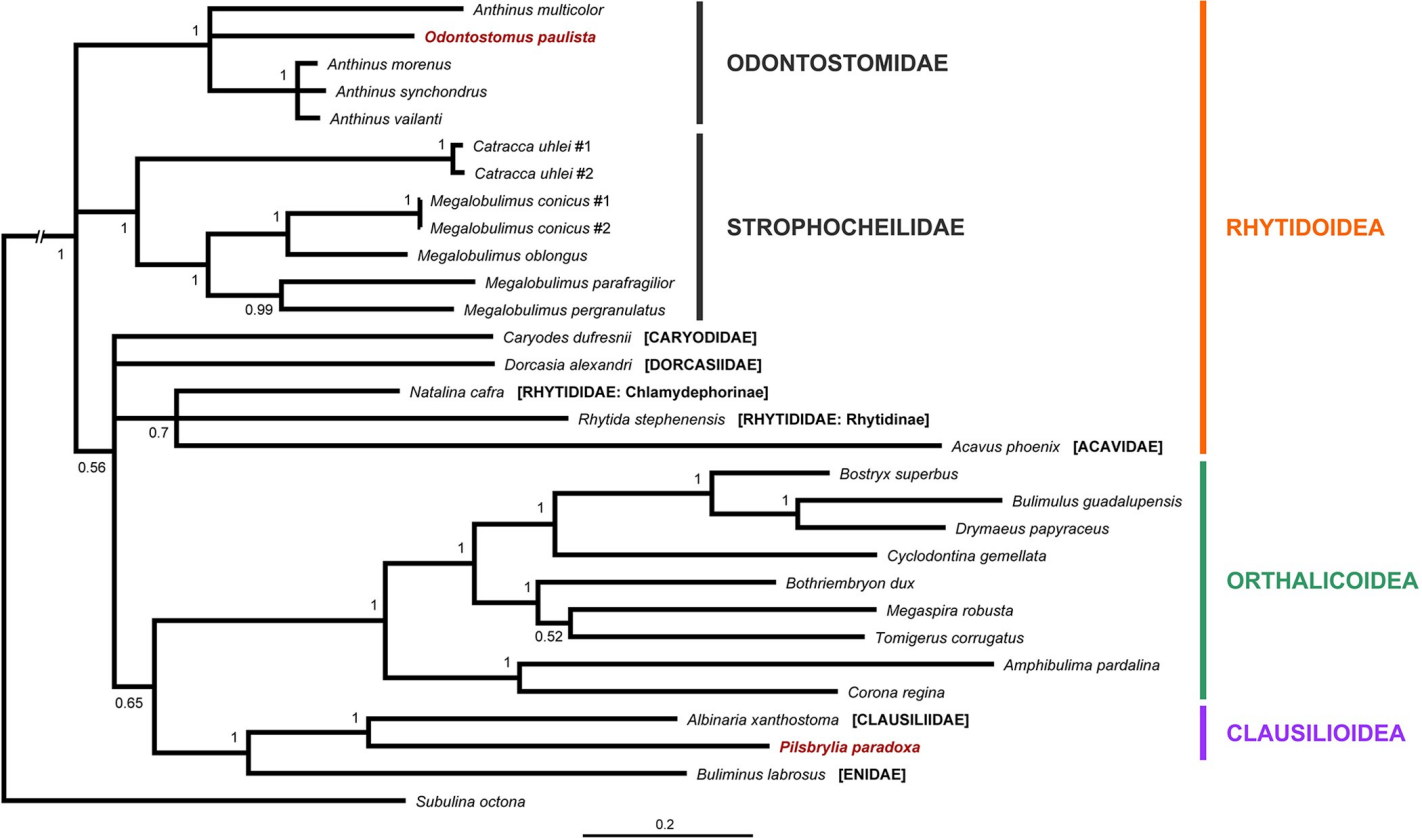
Bayesian inference tree showing the relationships of the genera *Odontostomus* and *Pilsbrylia*, which do not belong to Orthalicoidea. Posterior probabilities are shown on nodes. Scale bar is substitutions per site.

Most noticeably, in our phylogeny the genera *Odontostomus* and *Pilsbrylia* are placed outside of Orthalicoidea. Thus, an additional tree (with further species representing other stylommatophoran lineages) was built to further test their phylogenetic position. This second tree contained 30 terminal taxa, representing 28 species (Fig 7). The concatenated sequences (post trimming with Gblocks) included 1920 bp (COI: 641 bp; H3: 267 bp; ITS2+28S: 1012 bp).

Despite the polytomies on the family-level relationships of Rhytidoidea (Fig 7), the family groups with the taxa of interest are all maximally supported (PP = 1). Both *Odontostomus* and *Pilsbrylia* are found not to be members of Orthalicoidea. The former genus clusters with species of *Anthinus* (Strophocheilidae) in a polytomy, while the latter is sister to a representative of the Clausiliidae (PP = 1 in both cases).

Strophocheilidae (PP = 1) is resolved as paraphyletic. Rhytidoidea is also found to be paraphyletic but recent studies have shown that this superfamily (Rhytidoidea sensu lato) is presently an arrangement of convenience rather than an actual monophyletic lineage (e.g., [29]). Considering that Strophocheilidae is presently classified within Rhytidoidea sensu lato [2], we provisionally interpret Odontostomidae as also belonging to this group; however, further studies are needed to clarify the classification of Odontostomidae and Strophocheilidae.

### Orthalicoidea

The superfamily Orthalicoidea is a maximally supported clade (PP = 1; Figs 1 and 7). It is composed of two main clades: a maximally supported clade containing two families, Orthalicidae and Amphibulimidae (PP = 1), and a weakly supported clade containing all other families (PP = 0.69).

In the clade (Orthalicidae + Amphibulimidae), both families are monophyletic although without sufficient nodal support (PP = 0.55 each; Figs 1 and 2). However, the relationships within Orthalicidae remained essentially unresolved while those in Amphibulimidae were partly poorly supported. Most genera that were represented by more than one species were recovered as monophyletic (PP = 1, except for *Orthalicus*: PP = 0.92). The exception was *Plekocheilus*, which decayed into two deeply divergent clades, one of these containing *P*. *nebulosus*. However, *Plekocheilus* is a rather speciose genus, and an increased taxon sampling is needed before systematic conclusions should be drawn. The semi-slugs *Amphibulima* and the slugs *Gaeotis* and *Pellicula* cluster together in a polytomy, albeit without sufficient nodal support (PP = 0.54).

The second main clade, containing the rest of the Orthalicoidea, lacks sufficient nodal support (PP = 0.69). In this clade, *Paeniscutalus crenellus* forms the sister group of all remaining Orthalicoidea. However, the monophyly of these remaining Orthalicoidea is maximally supported (P = 1). In this clade, *Megaspira* is the sister lineage of all other taxa; a position that renders Megaspiridae as currently delineated non-monophyletic (Figs 1 and 3). The successively next more derived offshoot containing *Thaumastus* and *Kora* is also not well supported (PP = 0.68).

The sister group to the “Megaspiridae” clade *Thaumastus*+*Kora* is strongly supported (PP = 0.99). It contains a well-supported family-level clade (PP = 0.99), sister to the remainder Orthalicoidea, that includes the genus *Anostoma* as sister to a clade formed by *Hyperaulax* and *Tomigerus* (Figs 1 and 3), all of which are strongly supported (PP = 1). This clade is an assemblage of “orphaned” taxa that were previously classified in Odontostomidae and is herein recognized as a new family within Orthalicoidea. The name Tomogeridae is available for this clade (see Discussion below).

The next node (PP = 0.97) has Bothriembryontidae (PP = 1; Figs 1 and 4) as sister to the remainder Orthalicoidea. Interestingly, a species previously identified as *Rhinus taipuensis*, from northeast Brazil, is the sister taxon to all other bothriembryontids. *Rhinus* is member of the Simpulopsidae, so a new genus is described below to allocate this species (see below). In the clade comprising the remainder of Bothriembryontidae, there is a branch formed by the African *Prestonella* (PP = 1), sister to a polytomous clade that includes the South American *Plectostylus* and *Discoleus* and the bothriembryontids from Oceania. This renders the subfamily Prestonellinae paraphyletic. The Australasian Bothriembryontinae is monophyletic (though containing Placostylinae; PP = 0.94; Figs 1 and 4), containing the Australian *Bothriembryon* (recovered as paraphyletic) and a well-supported Pacific clade (PP = 1). The latter contain one branch from the Solomon Islands (PP = 1, with both genera being polyphyletic) and a branch with low support (PP = 0.7) comprising species from Vanuatu, New Caledonia, and New Zealand.

### Crown Orthalicoidea

The crown Orthalicoidea is a maximally-supported clade (PP = 1; Fig 5), sister to Bothriembryontidae, restricted to the Americas and containing three families, one of which is the extremely diverse Bulimulidae.

The most basal node in crown Orthalicoidea has Simpulopsidae (PP = 1) as sister to the rest. Simpulopsidae contains the genera *Rhinus*, *Leiostracus* and *Simpulopsis* (Fig 5); the latter two are non-monophyletic. The type species of *Leiostracus* (*L*. *vittatus*) is represented in our tree and its clade apparently excludes at least *L*. *perlucidus* from the genus. The type species of *Simpulopsis* (*S*. *sulculosa*) is also represented, belonging to a clade in a derived position, far removed from other supposed *Simpulopsis* spp.

The sister clade to Simpulopsidae (PP = 1) is formed by Bulimulidae and a new family. This new family has good support (PP = 0.95) and is composed of several genera that are “orphans” of Odontostomidae (Fig 5). There are no names available for this clade, so it is described below as Cyclodontinidae fam. nov. This family is seemingly composed of two main branches: the first containing the genera *Clessinia* and the monotypic *Ventania*, and the second containing the remaining genera. Within *Clessinia*, there are species that are not monophyletic. In the second branch, the genera *Plagiodontes* and *Bahiensis* are well supported (PP = 1), while *Cyclodontina* is polyphyletic and includes both *Moricandia* and a polyphyletic *Burringtonia*.

The final clade is a weakly supported Bulimulidae (PP = 0.80), although support is much stronger (PP = 0.95) after the first internal node (Fig 6; sister group to the clade *Otostomus* + *Cochlorina*). The relationship between the other more-encompassing monophyletic clades (of subfamily level) within Bulimulidae is more uncertain, although most of them have good support (Fig 6). The first of these groups is formed by the genera *Anctus*, *Oxychona* and *Leiostracus carnavalescus* (PP = 1). The latter clearly does not belong to *Leiostracus* (Simpulopsidae), and a new genus is erected to house it (see below).

The next group in the Bulimulidae is the subfamily Bostrycinae (PP = 1), composed solely of the genus *Bostryx* in a strict sense, as it contains the type species *B*. *solutus*. The genus, as understood until the present, is a wastebasket taxon and further studies will show if many of its species must now be either classified elsewhere or whether some of the multitude of synonyms for *Bostryx* [31] will need to be elevated to genus level within this subfamily. The final two groups are potentially sister taxa (unsupported, PP = 0.54; Fig 6) and consist in the subfamilies Bulimulinae and Peltellinae.

Bulimulinae is strongly supported (PP = 1) and includes the genera *Naesiotus*, *Protoglyptus*, *Rabdotus*, and *Bulimulus*, as well as a few species previously classified in *Bostryx*. The genera *Naesiotus and Protoglyptus* as currently understood are polyphyletic and include species previously classified in *Bostryx*, but the other genera are monophyletic (PP = 1). *Bulimulus tenuissimus*, often thought to be a species complex in southern Brazil, forms a tight clade with virtually no genetic distance (Fig 6).

Peltellinae is largely unsupported (PP = 0.70), so this group could represent a non-monophyletic assemblage. In our results, however, this subfamily contains the genera *Scutalus*, *Pseudoxychona*, *Neopetraeus*, and *Drymaeus*. *Drymaeus*, long thought to be a wastebasket taxon like *Bostryx*, is potentially monophyletic, pending the exclusion of *D*. *vexillum* and the inclusion of the slug genus *Peltella*. Inside *Drymaeus*, there are three distinct clusters representing some of its purported subgenera: (1) a clade with moderate support (PP = 0.94) containing *D*. *elongatus*, type species of subgenus *Mesembrinus*; (2) a strongly supported clade (PP = 1) containing *D*. *inusitatus*, type species of *Antidrymaeus*; and (3) a clade with low support (PP = 0.70) that likely represent nominate *Drymaeus*, even though its type species, *D*. *hygrohylaeus* (d’Orbigny, 1835), is absent. *Mesembrinus* and *Antidrymaeus* can thus be elevated to generic status (see below).

## Systematics

### Superfamily Orthalicoidea

**Cyclodontinidae fam. nov. Salvador & Breure. ZooBank reg. nr.:** urn:lsid:zoobank.org:act:4321653E-2A2F-4E52-8DC3-FE9AAC6FD6ED

**Type genus:** *Cyclodontina* Beck, 1837.

**Contained genera:** *Bahiensis* Jousseaume, 1877, *Burringtonia* Parodiz, 1944 [= *Pantagruelina* Forcart, 1946], *Clessinia* Doering, 1875 [= *Scalarinella* Dohrn, 1875; *Euodontostomus* Holmberg, 1912; *Spixia* Pilsbry & Vanatta, 1898; *Spixinella* Hylton Scott, 1952], *Cyclodontina* H. Beck, 1837, *Moricandia* Pilsbry & Vanatta, 1898, *Plagiodontes* Doering, 1876, *Ventania* Parodiz, 1940.

**Diagnosis:** Shell typically medium-sized, elongated fusiform. Teleoconch smooth or sculptured by axial ribs or irregular striae. Aperture typically displaying a columellar lamella plus a varying number (that can be zero) of teeth and knobs. Peristome typically reflexed, often thickened. Parietal callus sometimes thickened.

**Discussion:** The family Cyclodontinidae Salvador & Breure, fam. nov. is here established, containing a monophyletic assemblage of genera that were “orphaned” from Odontostomidae. The new family is understood as the sister taxon to the Bulimulidae (Fig 5). The taxon chosen as type genus was *Cyclodontina* (type species *C*. *inflata* (Wagner, 1827)), which is the oldest available generic name within the family.

#### Family Bothriembryontidae. Genus *Alterorhinus* gen. nov. Salvador, Silva & Cavallari

**ZooBank reg. nr.:** urn:lsid:zoobank.org:act:A0F89E89-1522-462C-A7A8-A42F47F2B9E9

**Type species:** *Bulimulus* (*Rhinus*) *rochai taipuensis* Baker, 1914 = *Alterorhinus taipuensis* (Baker, 1914) comb. nov.

**Contained species:** *Alterorhinus constrictus* (Pfeiffer, 1841) comb. nov., *Alterorhinus ovulum* (Reeve, 1849) comb. nov., *Alterorhinus rochai* (Baker, 1914) comb. nov., *Alterorhinus suturalis* (Baker, 1914) comb. nov., *Alterorhinus taipuensis* (Baker, 1914) comb. nov.

**Etymology:** From ‘alter’ (Latin for ‘alternate, other’) and *Rhinus*, the genus where the species were previously classified. The connective particle ‘o’ is used to aid in pronunciation. Grammatical gender: masculine.

**Diagnosis:** Shell bulimoid, multiwhorled, relatively slender to wide (expanded body whorl), with low whorls and well-marked suture. Protoconch (~1½ whorl) sculptured with fine wrinkled (zig-zag appearance) granulated striae that become less regular and increasingly granulated towards end of protoconch; first ½ whorl of protoconch may appear initially smooth; transition to teleoconch clearly marked. Aperture ovate-elongated; peristome reflexed, including the columellar region over the umbilicus. Thin columellar fold may be present.

**Discussion:** The present phylogeny places the specimens of *Rhinus taipuensis* as the sister taxon to all other Bothriembryontidae (Figs 1 and 4), far removed from *Rhinus* in Simpulopsidae. As such, the new genus *Alterorhinus* is proposed here to house it. Furthermore, other species previously classified in *Rhinus* [32] are here transferred to this new genus, considering that they (1) share a very similar shell morphology to the type species, including the diagnostic characters [31,33–36]; (2) have been considered related to the type species [34]; and (3) do not present any of the diagnostic morphological features of *Rhinus* (i.e., wide rotund shell, more fragile shell walls, periostracal hairs, a white spiral line on the medial region of the whorl).

*Alterorhinus constrictus* is known from Colombia, Venezuela, Guyana, and the Brazilian state of Roraima [31,33,35–39]. The records of *A*. *constrictus* from northeastern Brazil [32,40] are likely mistaken, belonging instead to one or more of its congeners, which are all restricted to that geographical area. *A*. *ovulum* is known only from Pernambuco state [32]; *A*. *rochai* is known from Pará to Pernambuco states in Brazil [32]; *A*. *suturalis* is known from Ceará to Bahia states [32,41]; and *A*. *taipuensis* is known from Ceará and Rio Grande do Norte states [32]. Therefore, the genus is distributed from Colombia to northeastern Brazil.

#### Family Bulimulidae. Genus *Sanniostracus* gen. nov. Salvador, Silva & Cavallari

**ZooBank reg. nr.:** urn:lsid:zoobank.org:act:CBB507A9-BEB8-472B-87D3-A4471F6D7429

**Type species:** *Leiostracus carnavalescus* Simone & Salvador, 2016 = *Sanniostracus carnavalescus* (Simone & Salvador, 2016) comb. nov.

**Contained species:** *Sanniostracus carnavalescus* (Simone & Salvador, 2016).

**Etymology:** From ‘sannio’ (Latin for ‘harlequin’) and ‘ostrakon’ (Greek for ‘shell’), referring to the typical red, white, and black coloration of the shell of the type species. Grammatical gender: masculine.

**Vernacular name:** harlequin snails.

**Diagnosis:** Shell bulimoid, medium-sized, multi-whorled (whorls growing regularly in size), with acuminated apex. Shell base colour white, but overall colour pattern widely varied, with multiple combinations of solid spiral lines and dotted spiral lines in the colours red, orange, yellow, brown, and black. Protoconch (~1½ whorl) sculptured by fine closely-packed sinuous axial riblets in upper portion and numerous fine spiral striae in lateral portion; transition to teleoconch clear. Whorl profile slightly convex; keel absent. Aperture medium-sized, oval. Peristome reflexed, partially covering narrow umbilicus.

**Discussion:** The present phylogeny places specimens of *Leiostracus carnavalescus* within Bulimulidae, in a clade containing representatives of the genera *Oxychona* (sister taxon) and *Anctus* (Fig 6), far removed from *Leiostracus* in Simpulopsidae. As such, the new genus *Sanniostracus* is proposed here to house it.

In their study describing *Sanniostracus carnavalescus* [42], the authors noted the morphoanatomical similarities between their new species and member of the family Bulimulidae. Nevertheless, other similarities with *Leiostracus* spp. led them to classify their species in the latter genus. Under the light of the present phylogeny, those can now be interpreted as superficial similarities (e.g., shell shape, black and white body pattern) and the differences to *Leiostracus* become clearer, the most prominent of which is the protoconch sculpture, which in *Leiostracus* consists of sinuous subsutural axial riblets that give way to spiral cordlets towards the median region of the whorl [31,43].

Likewise, the shared characters between *Sanniostracus* and *Oxychona* become starkly clear, even though the species in the latter genus all have wide conical and strongly keeled shells. The soft body is of similar shape, white in colour, with a wide black band on each side of the head, positioned immediately below the eye stalks and that can end in the median region of the foot or extend itself toward the “tail” [43,44]. The base colour of shells is white, and the colour pattern has variable solid and dotted lines of multiple earth-toned colours [32,43,44]. The radular teeth are similar in shape, with a small blunt rachidian tooth, spatula-like lateral teeth, and bicuspid marginal teeth bearing small acute ectocones posteriorly positioned at their base [31,42].

**Remarks:** *S*. *carnavalescus* is highly polymorphic in shell colour patterns [42]. However, the holotype and paratype (red morph) show a reasonable genetic distance between them (Fig 6), which might be indicative that they potentially represent two different sympatric taxa. Further molecular investigation using a series of additional specimens (also including the black morph; [42]) is advised.

## Discussion

The results of the present phylogenetic analysis allow that several modifications (some major, some minor) be made to the classification of the Orthalicoidea. These are explained in the section that follows. Furthermore, the new framework also brings new biogeographical insights and has implications on how to interpret the fossil record; these topics are discussed in detail further below.

### Classification

Here are delineated all the implications the present study has for the phylogeny of the Orthalicoidea. As in the Results section above, the taxa are treated in the order of the nodes starting from the root (Figs 1–6).

#### Pilsbrylia

The genus *Pilsbrylia* contains three species found in southeastern Brazil and northern Argentina [45]. While it bears superficial similarity in shell shape to crown orthalicoid genera such as *Cyclodontina* and *Clessinia*, our results showed that it does not belong in Orthalicoidea (Figs 1 and 4), as already suggested by [7]. Instead, it is closely related to the door-snails (Clausiliidae). While clausiliids haver narrow turreted shells of which *Pilsbrylia* is reminiscent, there is a major difference: the shells of door-snails are sinistral, while *Pilsbrylia* is dextral like the majority of the Stylommatophora. The exact placement of this genus in relation to the door-snails needs to be further investigated, particularly if it will cluster with South American Clausiliidae.

#### Odontostomidae

The representative of *Odontostomus* in our analyses forms a group with the Strophocheilidae genus *Anthinus* in a polytomy with the remainder Strophocheilidae (Fig 7). The Strophocheilidae are paraphyletic, pending the exclusion of *Anthinus*. To solve this, here we transfer the genus *Anthinus* to Odontostomidae; furthermore, we classify Odontostomidae within Rhytidoidea sensu lato.

This leaves the former “odontostomid” genera in Orthalicoidea without a family name. Those genera are divided into two unrelated branches within Orthalicoidea (see the entries below for Tomogeridae and Cyclodontinidae fam. nov.).

We have sequenced a single species of *Odontostomus* (*O*. *paulistus*) in the present analysis, which is not the type species of the genus (*O*. *odontostomus* (Sowerby, 1824)). Nevertheless, based on close morphological similarity we are confident that *O*. *paulistus* is a congener of *O*. *odontostomus* and thus, a good representative of the genus and family. We are likewise confident that it belongs in a different group than the Orthalicoidea, even though this result might seem unexpected at first sight. The shells of *Odontostomus* are unlike any other Orthalicoidea, including those previously classified as odontostomids. Rather, *Odontostomus* have sturdy shells reminiscent of Strophocheilidae and particularly similar to *Anthinus* [32,46]. In particular, the shells are medium to large sized, with strong walls, often with bulging whorls, a coarse teleoconch sculpture, a thick expanded peristome that typically covers the umbilicus, and an ochre to brown periostracum bearing in some cases an “camouflaged” pattern. The shells of members of the Strophocheilidae genus *Gonyostomus* (of which *Anthinus* was a subgenus) also share these features [32,46] and is, therefore, likewise here transferred to Odontostomidae.

Ongoing molecular studies by members of our group focusing on the Strophocheilidae will now also include the Odontostomidae to further investigate the relationship of these two families (and other Rhytidoidea sensu lato). Hopefully we will be able to elucidate if they are sister taxa, consecutive branches of the Rhytidoidea, or even synonymous. A recent morphological analysis [46] has placed *Anthinus* inside Strophocheilidae, but it did not include *Odontostomus* or *Gonyostomus*.

#### Orthalicidae & Amphibulimidae

Each of these families have no support, though together they form a strongly supported clade (Figs 1 and 2). As such, there is the possibility that one or both families are not monophyletic and further studies focusing on them are necessary. If that is confirmed, Amphibulimidae should be considered part of (and thus a synonym) of Orthalicidae. Breure & Romero [7] also recovered these families as sister taxa in the base of Orthalicoidea, although a monophyletic Orthalicidae was likewise unsupported.

By the confusion within the genus *Orthalicus* (Fig 2), it can be seen that this genus would benefit from a revision. Likewise, the paraphyletic genus *Plekocheilus* is also in need of a revision, and some subgenera (and/or synonymized names) might need to be elevated to genera. The Paleogene monotypic genus *Cortana* from Rio de Janeiro, Brazil, was interpreted to be related to *Eudolichotis* Pilsbry, 1896 (presently considered a synonym of *Plekocheilus* and represented by *P*. *lacerta* in our phylogeny; Fig 2), but classified in the Bulimulidae [4,47]. Thus, *Cortana* is here transferred to Amphibulimidae.

The semi-slugs *Amphibulima* and the slugs *Gaeotis* and *Pellicula* seem to form a monophyletic group, although with no support, with a polytomy (Fig 2). This group, however, makes sense from an evolutionary perspective, considering that it is more parsimonious that the shell was reduced only once and then internalized in *Gaeotis* and *Pellicula*, which might have happened once or twice depending on how the polytomy is solved. Furthermore, the species *Gaeotis flavolineata*, which is typically considered a junior synonym of *G*. *nigrolineata* (e.g., [48]), was shown to be genetically distant from the latter (Fig 2). This result supports *G*. *flavolineata* as a distinct species.

#### “Megaspiridae”

Despite this being a paraphyletic assemblage (Figs 1 and 3), it remains a useful group, so we will retain it here for stability. This result is not unexpected, as this family unites species with very distinct shell morphologies (most notably the turreted *Megaspira*). Breure & Romero [7] observed a monophyletic Megaspiridae, albeit with low support, and suggested that this group was a basal relict group of Orthalicoidea, which is not exactly the case given the position of Orthalicidae + Amphibulimidae (Fig 1).

We confirmed the placement of *Paeniscutalus* within this family, as provisionally suggested by [49]. The eastern Brazilian genus *Kora*, previously classified in Bulimulidae [50], is shown here to be closely related to *Thaumastus*, and is thus transferred to family “Megaspiridae”.

#### Tomogeridae

This branch is one of the two monophyletic groups in the phylogeny that is an “orphan” of Odontostomidae, where its genera and species were previously classified (Figs 1 and 3). [7] had no representatives of this group in their study.

Given its position in the tree, this clade should be recognized as a family-level taxon. Fortunately, there is already a name available in the literature for it: Tomogeridae Jousseaume, 1877. Jousseaume [51] proposed the family among the “*Bulimus*” to allocate the genera *Tomogeres* Montfort, 1810 and *Tomogerina* Jousseaume, 1877 (both synonyms of *Anostoma* Fischer von Waldheim, 1807).

The genus *Anostoma*, despite its strikingly different morphology with an apically bent body whorl, was later conflated with other Orthalicoidea whose shells had apertural barriers (the Odontostomidae, as understood until now). The present molecular phylogeny supports Jousseaume’s assertion [51] that *Anostoma* lineage represents a separate and unique family. Our study also includes two other genera in this family: *Tomigerus* Spix, 1827 and the island endemic *Hyperaulax* Pilsbry, 1897. The species in both genera have similarities in their shells that indicated their affinity and their sister-taxa relationship proposed by [10] is fully supported here (Fig 3). Likewise, we propose here that the genera *Biotocus* Salgado & Leme, 1990 and the cave endemic *Clinispira* Simone & Casati, 2013, which have shells closely resembling *Tomigerus* [32,52,53], also belong in this resurrected family.

Bouchet et al. [2] argued that a petition to the ICZN was necessary to conserve the younger name Odontostomidae Pilsbry & Vanatta, 1898 over Tomogeridae Jousseaume, 1877. However, that is not necessary, as it has been shown here that the two families are two completely distinct lineages (see also the entry for Odontostomidae above).

#### Bothriembryontidae

Bothriembryontidae is monophyletic (Figs 1 and 4), and its branches closer to the family’s basal node comprise South American and African species: the non-monophyletic subfamily “Prestonellinae”, which we maintain here because it is a useful grouping. The subfamily Bothriembryontinae is paraphyletic pending the including of Placostylinae; as such, here we consider Placostylinae a synonym of Bothriembryontinae.

The paraphyletic Prestonellinae was also observed in [7], although those authors had monophyletic Bothriembryontinae and Placostylinae (with low support), likely due to the smaller sample of taxa.

The presence of this family in South America was until now considered relict, with just the genera *Plectostylus* and *Discoleus* in the southern and western regions of the continent. However, *Alterorhinus* gen. nov. was revealed to be the sister taxon to all other Bothriembryontidae; its species presently inhabit a large area in northern and eastern South America, showing that the family is widespread in the continent and hinting that it was potentially more diverse in the past.

The Australian genus *Bothriembryon*, as currently understood, is not monophyletic and would benefit from a revision. The two genera from the Solomon Islands, *Eumecostylus* and *Placocharis* are polyphyletic. Considering that there are no consistent diagnostic characters separating these two genera [54], here we consider *Placocharis* a junior synonym of *Eumecostylus*.

*Maoristylus* (from New Zealand and Lord Howe Island) has been considered either as a subgenus of New Caledonian *Placostylus* or its synonym, but it was recently shown to be a distinct genus [55]. These two genera have thus been considered as sister taxa, albeit in works with low taxon coverage (e.g., [56]). The inclusion of Vanuatuan *Diplomorpha* in the present study has shown that is not necessarily the case, as it clusters (with low support) with the New Caledonian *Placostylus*.

#### Simpulopsidae

This is a well-supported family and most of its internal relationships are resolved with good support (Fig 5). This result confirms the findings of [7], who redefined the family Simpulopsidae and established it as the sister to the remainder crown Orthalicoidea.

Still, the genera *Leiostracus* and *Simpulopsis* are not monophyletic and need a thorough revision. Two further genera, even though not represented in our study, likely belong in this family as well: *Lopesianus* Weyrauch, 1958 and *Eudioptus* Martens, 1860 (see discussion in [57]). Simpulopsidae is neotropical, with only a few species present in the sub-tropical areas of South America (e.g., [32,58]).

#### Cyclodontinidae fam. nov.

This newly established family has a somewhat resolved internal structure, although most clades have low support (Fig 5). While some genera were recovered as monophyletic (*Clessinia*, *Plagiodontes* and *Bahiensis*), the type genus *Cyclodontina* as presently understood is polyphyletic, including the genera *Moricandia* and a likewise polyphyletic *Burringtonia*. The type species of *Cyclodontina*, *C*. *inflata*, could not be included in our analysis; *C*. *inflata* is conchologically distinct from other congeners present in the tree, so we cannot speculate where it would be placed. As such, the restructuring of the genus *Cyclodontina* (and *Moricandia* and *Burringtonia*) will remain for a future study. Cyclodontinidae is endemic to South America and restricted to Bolivia, Brazil, Paraguay, Uruguay, and Argentina [49,54].

Notably, [7] recovered a similar clade (they named it Odontostomidae, following the then current practice and considering that they did not have a representative of *Odontostomus*), albeit with the genus *Bahiensis* being place outside of it and as sister taxon to Bulimulidae. In our tree (Fig 5), with a larger sampling, *Bahiensis* is placed within Cyclodontinidae; its long branch is likely due to the lack of COI sequences for both its species (Table 2).

#### Bulimulidae

The Bulimulidae are weakly supported when considering that it includes the branch formed by *Otostomus* and *Cochlorina* (Fig 6), two genera defined by their shell’s barrel-like body whorl and highly modified aperture. Given their morphological similarities (e.g., [32]), their close relationship in the phylogeny makes immediate sense. Excluding this branch, the remainder of Bulimulidae is well supported (Fig 6).

The first branch includes *Anctus*, *Oxychona* and *Sanniostracus* gen. nov. As shown above, despite the obvious difference in shell shape, the latter two share many conchological and morphoanatomical features. The next branch of Bulimulidae is the subfamily Bostrycinae, containing only the newly circumscribed genus *Bostryx*.

The Bulimulinae includes *Naesiotus*, *Protoglyptus*, *Rabdotus*, and *Bulimulus*. *Naesiotus* and *Protoglyptus*, as currently understood, are non-monophyletic and need further investigation. We could not include their type species *Naesiotus nux* (Broderip, 1832) and *Protoglyptus pilosus* (Guppy, 1871) in our analysis, so the actual placement and composition of these genera remain uncertain. Nevertheless, considering that we can better define *Bostryx* based on its type species as explained above, we preliminarily transfer some of those “*Bostryx*” spp. within Bulimulinae to the genus *Naesiotus* (Table 4).

**Table 4:** Summary of species transferred to other genera in the present study, followed by their new combinations.

Previous classification	New classification (this study)
*Bostryx apodemetes* (d’Orbigny, 1835)	*Naesiotus apodemetes* (d’Orbigny, 1835) comb. nov.
*Bostryx bilineatus* (G.B. Sowerby I, 1833)	"*Bostryx*" *bilineatus* (G.B. Sowerby I, 1833)
*Bostryx strobeli* Parodiz, 1956	*Naesiotus strobeli* (Parodiz, 1956) comb. nov.
*Drymaeus dormani* (W.G. Binney, 1857)	*Antidrymaeus dormani* (W.G. Binney, 1857) comb. nov.
*Drymaeus elongatus* (Röding, 1789)	*Mesembrinus elongatus* (Röding, 1789) comb. nov.
*Drymaeus expansus* (L. Pfeiffer, 1848)	"*Drymaeus*" *expansus* (L. Pfeiffer, 1848)
*Drymaeus gereti* C.M.F. Ancey, 1901	*Antidrymaeus gereti* (C.M.F. Ancey, 1901) comb. nov.
*Drymaeus immaculatus* (C.B. Adams, 1850)	*Antidrymaeus immaculatus* (C.B. Adams, 1850) comb. nov.
*Drymaeus inusitatus* (Fulton, 1900)	*Antidrymaeus inusitatus* (Fulton, 1900) comb. nov.
*Drymaeus laticinctus* (Guppy, 1868)	*Antidrymaeus laticinctus* (Guppy, 1868) comb. nov.
*Drymaeus multifasciatus* (Lamarck, 1822)	*Antidrymaeus multifasciatus* (Lamarck, 1822) comb. nov.
*Drymaeus multilineatus* (Say, 1825)	*Mesembrinus multilineatus* (Say, 1825) comb. nov.
*Drymaeus rufescens* (J.E. Gray, 1825)	*Antidrymaeus rufescens* (J.E. Gray, 1825) comb. nov.
*Drymaeus stramineus* (Guilding, 1824)§	*Antidrymaeus stramineus* (Guilding, 1824) comb. nov.
*Drymaeus sulphureus* (L. Pfeiffer, 1857)	*Antidrymaeus sulphureus* (L. Pfeiffer, 1857) comb. nov.
*Drymaeus vexillum* (Broderip, 1832)	"*Drymaeus*" *vexillum* (Broderip, 1832)
*Leiostracus carnavalescus* Simone & Salvador, 2016	*Sanniostracus carnavalescus* (Simone & Salvador, 2016) comb. nov.
*Leiostracus faerie* Salvador & Cavallari, 2014	*Pseudoxychona faerie* (Salvador & Cavallari, 2014) comb. nov.
*Naesiotus subcostatus* (Haas, 1948)	"*Naesiotus*" *subcostatus* (Haas, 1948)
*Peltella iheringi* Leme, 1968	*Drymaeus iheringi* (Leme, 1968) comb. nov.
*Peltella palliolum* (Férussac, 1821)	*Drymaeus palliolum* (Férussac, 1821) comb. nov.
*Placocharis macfarlandi* (Brazier, 1876)	*Eumecostylus macfarlandi* Brazier, 1876
*Placocharis malaitensis* (Clench, 1941)	*Eumecostylus malaitensis* (Clench, 1941) comb. nov.
*Placocharis ophir* (Clench, 1941)	*Eumecostylus ophir* (Clench, 1941) comb. nov.
*Rhinus constrictus* (L. Pfeiffer, 1841)	*Alterorhinus constrictus* (L. Pfeiffer, 1841) comb. nov.
*Rhinus ovulum* (Reeve, 1849)	*Alterorhinus ovulum* (Reeve, 1849) comb. nov.
*Rhinus rochai* (F. Baker, 1914)	*Alterorhinus rochai* (F. Baker, 1914) comb. nov.
*Rhinus suturalis* (F. Baker, 1914)	*Alterorhinus suturalis* (F. Baker, 1914) comb. nov.
*Rhinus taipuensis* (F. Baker, 1914)	*Alterorhinus taipuensis* (F. Baker, 1914) comb. nov.

Peltellinae contains the genera *Scutalus*, *Pseudoxychona*, *Neopetraeus* and *Drymaeus*. The close relationship of *Pseudoxychona* to *Drymaeus* was expected considering their similarly reticulated protoconchs, despite the similarities of the shells between *Pseudoxychona* and *Leiostracus* (Simpulopsidae). Considering this new finding, one species previously described by two of the present authors [59] is herein transferred to *Pseudoxychona*: *Pseudoxychona faerie* (Salvador & Cavallari, 2014) comb. nov.

*Drymaeus* is potentially monophyletic, though it might need the exclusion of a few species such as *D*. *vexillum*. A revision of this genus with more representatives (and its type species, *D*. *hygrohylaeus* (d’Orbigny, 1835)) is advised, considering that some of its subgenera and/or synonymized names (e.g., *Leptodrymaeus*) might have phylogenetic reality and help to achieve a better classification. For instance, the monophyletic clades representing subgenera *Mesembrinus* (type species *D*. *virgulatus*) and *Antidrymaeus* (type species *D*. *inusitatus*) are elevated here to the genus level (Fig 6).

The genus *Peltella* of bright-colored slugs is contained within *Drymaeus* (as already suggested by [7]) and thus considered its synonym. It is an interesting case of limacization; only two instances of such phenomenon occurred in Orthalicoidea (the other being the Amphibulimidae clade *Amphibulima* + *Gaeotis* + *Pellicula*; Fig 6).

The relationships between Bostrycinae, Bulimulinae and Peltellinae are unclear, as the support values are low (even though the subfamilies Bostrycinae and Bulimulinae are each strongly individually supported). Overall, they have a similar arrangement to the Bulimulidae recovered by [7], although the sister-taxon relationship between the groups varies. Of the three subfamilies, Peltellinae has the lowest support (including its inner nodes), which hints at a potential non-monophyletic group. In any event, it is uncertain whether a division in subfamilies is really needed in Bulimulidae, particularly considering that Bostrycinae is monotypic at present. Furthermore, that could raise the need of the other branches to be recognized as further subfamilies, which would cause unnecessary taxonomic inflation.

**Vidaliellidae:** As mentioned above, the placement of the extinct Vidaliellidae in the Orthalicoidea [3] was based on superficial and incomplete conchological comparison and without fully considering the biogeographical history and chronology of the taxa involved.

The size and shape of the shells, their multiwhorled spire that is comparatively short when compared to the expanded body whorl, the aperture shape, the strongly reflexed peristome, and the presence of a strong parietal callus, are all features shared with the strong-shelled Rhytidoidea such as the southern African Dorcasiidae and Malagasy Clavatoridae (and even the South American Strophocheilidae) [54,60]. In particular, the fossils are strongly reminiscent of *Clavator* Martens, 1860 and *Leucotaenius* Martens, 1860, to the point that several Vidaliellidae species were previously classified in those genera ([3] and references therein).

The link to the Malagasy Clavatoridae was considered biogeographically unlikely by [3]; yet those authors suggested a classification in Orthalicoidea, which is even more unlikely, from biogeographical, chronological, and conchological standpoints. As such, here we provisionally classify the Vidaliellidae in the Rhytidoidea sensu lato. By considering Vidaliellidae part of an African lineage of rhytidoids instead of having a South American ancestry, the presence of this family in the Paleogene of northern Africa becomes less aberrant. The European fossils of Vidaliellidae, however, need to be reassessed. While some may in fact represent a branch of this family that extended into Europe when the climate was warmer [3], others seem more closely related to typical European taxa such as the Filholiidae (e.g., [61,62]).

### Paleobiogeography

The superfamily Orthalicoidea likely originated in South America and was already present there in the Late Cretaceous [4,5,7]. Notably, the family Bothriembryontidae is the only truly Gondwanan lineage known so far, with representatives in South America, southern Africa, and Australia (and later New Zealand and a few other Pacific Islands). Thus, the origin of Orthalicoidea might lie in older times in the Cretaceous.

However, the Cretaceous orthalicoid fossils need to be revised considering the new phylogenetic framework and classification proposed herein. Several of those fossils have been assigned to modern genera in crown Orthalicoidea such as *Bulimulus* and *Bahiensis* [4,5], but they likely belong to lineages that appeared earlier, such as Orthalicidae and “Megaspiridae”. For instance, the monotypic genus *Cortana* from the Late Paleocene [4,47] is now considered a member of the Amphibulimidae rather than Bulimulidae, as explained above.

By the Late Paleocene, all families of Orthalicoidea were already established, as evidenced by the fossils of Cyclodontinidae and Bulimulidae from Itaboraí Basin, Rio de Janeiro, Brazil [4,47]. In all likelihood, the different branches of this superfamily (Orthalicidae, Amphibulimidae, Simpulopsidae, and Bulimulidae) spread to Central America and the Caribbean long before the Isthmus of Panama formed around 3 million years ago [63] and physically connected the continents.

## Conclusion

**Fig 8:**
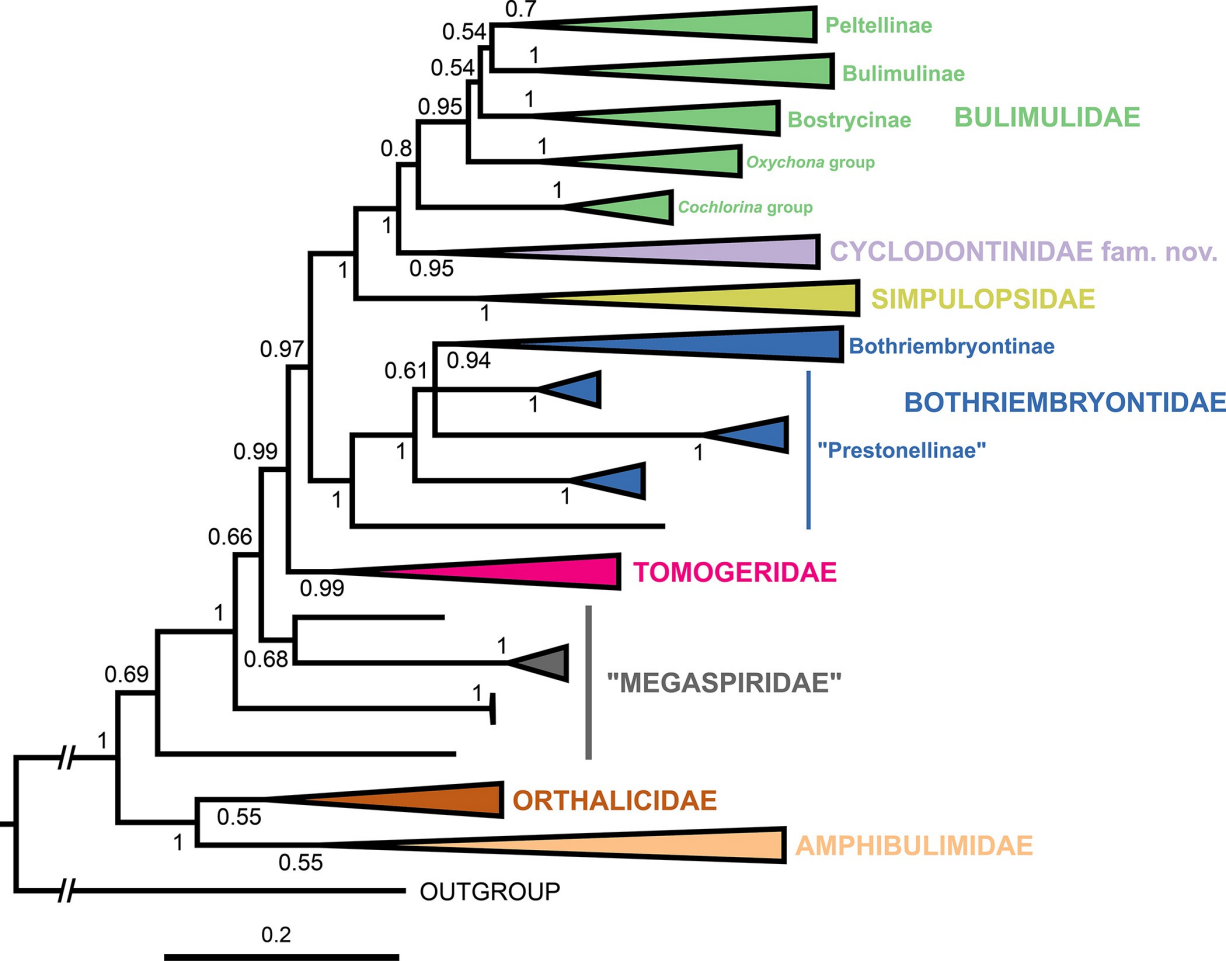
Summary tree of the Orthalicoidea, showing the family and subfamily level taxa. Bayesian posterior probabilities are shown on nodes. Scale bar is substitutions per site.

Based on the present results, we propose the following revised classification of superfamily Orthalicoidea Martens, 1860 (Fig 8). Quotation marks are used to indicate non-monophyletic (sub)family-level assemblages. We list only those genera that are represented in our analysis or that have been otherwise discussed above. A summary of species transferred to other genera and the new combinations can be seen in Table 4.

### Superfamily Orthalicoidea Martens, 1860

#### Family Amphibulimidae Fischer, 1873

*Amphibulima* Lamarck, 1805

*Cortana* Salvador & Simone, 2013

*Gaeotis* Shuttleworth, 1854

*Pellicula* Fischer, 1856

*Plekocheilus* Guilding, 1827

#### Family Bothriembryontidae Iredale, 1937

Subfamily Bothriembryontinae Iredale, 1937 [= Placostylinae Pilsbry, 1946]

*Bothriembryon* Pilsbry, 1894

*Diplomorpha* Ancey, 1884

*Eumecostylus* Martens, 1860 [= *Placocharis* Pilsbry, 1900]

*Maoristylus* Haas, 1935

*Placostylus* Beck, 1837

Subfamily “Prestonellinae” van Bruggen, Herbert & Breure, 2016

*Alterorhinus* gen. nov. Salvador, Silva & Cavallari

*Discoleus* Breure, 1978

*Plectostylus* Beck, 1837

*Prestonella* Connolly, 1929

#### Family Bulimulidae Tryon, 1867

Unranked Bulimulidae

*Anctus* Martens, 1860

*Cochlorina* Jan, 1830

*Otostomus* Beck, 1837

*Oxychona* Mörch, 1852

*Sanniostracus* gen. nov. Salvador, Silva & Cavallari

Subfamily Bostrycinae Breure, 2012

*Bostryx* Troschel, 1847

Subfamily Bulimulinae Tryon, 1867

*Bulimulus* Leach, 1814 [= *Cochlogena* Férussac, 1821; *Siphalomphix* Rafinesque, 1833]

*Naesiotus* Albers, 1850

*Protoglyptus* Pilsbry, 1897

*Rabdotus* Albers, 1850

Subfamily Peltellinae Gray, 1855

*Drymaeus* Albers, 1850 [= *Leptodrymaeus* Pilsbry, 1946; *Peltella* Gray, 1855]

*Antidrymaeus* Germain, 1907

*Mesembrinus* Albers, 1850

*Neopetraeus* Martens, 1885

*Pseudoxychona* Pilsbry, 1930

*Scutalus* Albers, 1850

**Family Cyclodontinidae fam. nov. Salvador & Breure.** *Bahiensis* Jousseaume, 1877

*Burringtonia* Parodiz, 1944 [= *Pantagruelina* Forcart, 1946]

*Clessinia* Doering, 1875 [= *Scalarinella* Dohrn, 1875; *Euodontostomus* Holmberg, 1912; *Spixia* Pilsbry & Vanatta, 1898; *Spixinella* Hylton Scott, 1952]

*Cyclodontina* Beck, 1837

*Moricandia* Pilsbry & Vanatta, 1898

*Plagiodontes* Doering, 1876

*Ventania* Parodiz, 1940

#### Family “Megaspiridae” Pilsbry, 1904

*Kora* Simone, 2012

*Megaspira* Lea, 1836

*Paeniscutalus* Wurtz, 1947

*Thaumastus* Martens, 1860

#### Family Orthalicidae E. von Martens, 1860 [= Liguidae Pilsbry, 1891]

*Corona* Albers, 1850

*Kara* Strebel, 1910

*Liguus* Montfort, 1810

*Orthalicus* Beck, 1837

*Porphyrobaphe* Shuttleworth, 1856

*Sultana* Shuttleworth, 1856

#### Family Simpulopsidae Schileyko, 1999

*Eudioptus* Martens, 1860

*Leiostracus* Albers, 1850

*Lopesianus* Weyrauch, 1958

*Rhinus* Martens, 1860

*Simpulopsis* Beck, 1837

#### Family Tomogeridae Jousseaume, 1877

*Anostoma* Fischer von Waldheim, 1807 [= *Tomogeres* Montfort, 1810; *Angystoma* Schumacher, 1817; *Tomogerus* Blainville, 1824; *Anastoma* Cristofori & Jan, 1832; *Ringicella* Gray, 1847; *Tomogerina* Jousseaume, 1877]

*Biotocus* Salgado & Leme, 1990

*Clinispira* Simone & Casati, 2013

*Hyperaulax* Pilsbry, 1897

*Tomigerus* Spix, 1827 [= *Digerus* Haas, 1937; *Pilsbryella* Ihering, 1905 *non* Nierstrasz, 1905; *Cearella* Ihering, 1906]

The family Vidaliellidae Nordsieck, 1986 is excluded from Orthalicoidea and transferred to the Rhytidoidea sensu lato. The genus *Pilsbrylia* Hylton Scott, 1952 is removed from Orthalicoidea and transferred to the Clausilioidea. It is herein considered as Clausilioidea *incertae sedis* until further research is conducted. The genus *Odontostomus* Beck, 1837 (and hence, the family Odontostomidae Pilsbry & Vanatta, 1898) is removed from Orthalicoidea and transferred provisionally to Rhytidoidea sensu lato. Family Odontostomidae contains the genera *Odontostomus* [= *Macrodontes* Swainson, 1840; *Macrodontopsis* Thiele, 1931], *Anthinus* Albers, 1850, and *Gonyostomus* Beck, 1837 [= *Gonyostoma* Swainson, 1840] (the latter two being thus removed from Strophocheilidae).

## Supporting information

S1 Fig Bayesian inference tree of the Orthalicoidea showing the complete set of terminal taxa. Species names are shown in regular font to facilitate visualization. Posterior probabilities are shown on nodes. Scale bar is substitutions per site.
(PDF)
