# Supplementary figures and images for: Molecular phylogeny of the Orthalicoidea land snails: Further support and surprises
Source: PLoS One. 2023 Jul 26;18(7):e0288533. doi: 10.1371/journal.pone.0288533 (PMC10370776; doi:10.1371/journal.pone.0288533)

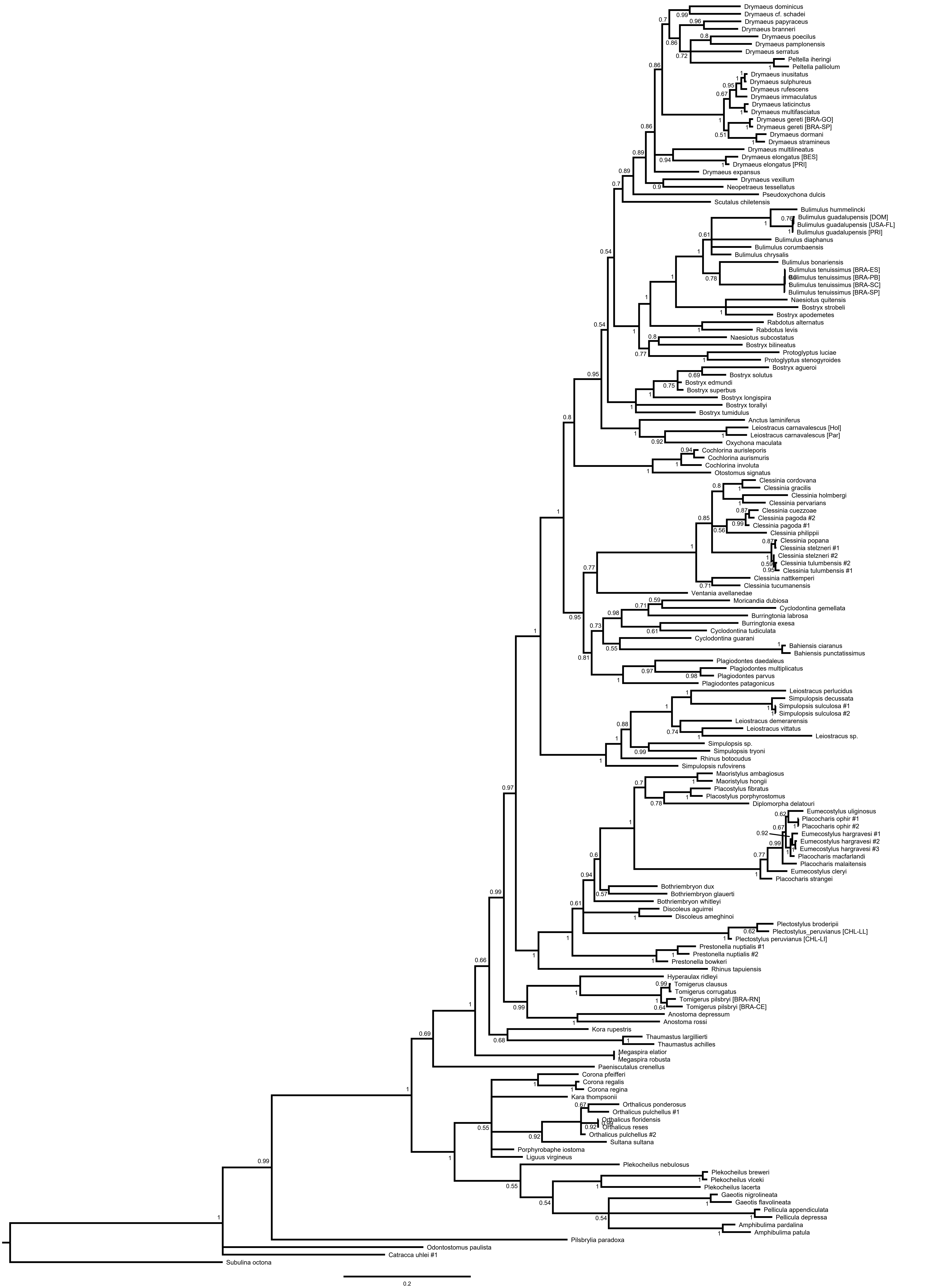

Supplement: S1 Fig — Species names are shown in regular font to facilitate visualization. Posterior probabilities are shown on nodes. Scale bar is substitutions per site. (PDF) [file pone.0288533.s001.pdf]
